# Precision parameter estimation in Proton Exchange Membrane Fuel Cells using depth information enhanced Differential Evolution

**DOI:** 10.1038/s41598-024-81160-0

**Published:** 2024-11-28

**Authors:** Pradeep Jangir, Absalom E. Ezugwu, Arpita, Sunilkumar P. Agrawal, Sundaram B. Pandya, Anil Parmar, G. Gulothungan, Laith Abualigah

**Affiliations:** 1 University Centre for Research and Development, Chandigarh University, Gharuan, Mohali, Punjab, 140413 India; 2 Department of CSE, Graphic Era Hill University, Dehradun, 248002 India; 3 Department of CSE, Graphic Era Deemed, To Be University, Dehradun, Uttarakhand 248002 India; 4 Jadara University Research Center, Jadara University, Irbid, Jordan; 5 Unit for Data Science and Computing, North-West University, 11, Hofman Street, Potchefstroom, 2520 South Africa; 6 Department of Biosciences, Saveetha School of Engineering, Saveetha Institute of Medical and Technical Sciences, Chennai, 602 105 India; 7 Department of Electrical Engineering, Government Engineering College, Gandhinagar, Gujarat India; 8 Department of Electrical Engineering, Shri K.J. Polytechnic, Bharuch, 392 001 India; 9 Department of Electronics and Communication Engineering, SRM Institute of Science and Technology, SRM Nagar, Kattankulathur, Chengalpattu, Tamilnadu 603203 India; 10 Computer Science Department, Al Al-Bayt University, Mafraq, 25113 Jordan; 11 Jadara University Research Center, Jadara University, PO Box 733, Irbid, Jordan; 12 Applied Science Research Center, Applied Science Private University, Amman, 11931 Jordan; 13 Centre for Research Impact & Outcome, Chitkara University Institute of Engineering and Technology, Chitkara University, Rajpura, 140401 Punjab India

**Keywords:** Parameter estimation, Proton Exchange Membrane Fuel Cell, PEMFC, Differential Evolution, Optimization, Energy storage, Fossil fuels, Fuel cells, Renewable energy

## Abstract

Proton Exchange Membrane Fuel Cell (PEMFC) models require parameter tuning for their design and performance improvement. In this study, Depth Information-Based Differential Evolution (Di-DE) algorithm, a novel and efficient metaheuristic approach, is applied to the complex, nonlinear optimization problem of PEMFC parameter estimation. The Di-DE algorithm was tested on twelve PEMFCs (BCS 500 W PEMFC, Nedstack 600 W PS6 PEMFC, SR-12 500 W PEMFC, H-12 PEMFC, STD 250 W PEMFC, HORIZON 500 W PEMFC and four 250W PEMFC and two H-12 12W PEMFC) and showed excellent accuracy. The Di-DE algorithm is was compared with other advanced evolutionary algorithms like iwPSO, CLPSO, DNLPSO, SLPSO, SaDE, SHADE, JADE, QUATRE, LSA, QUATRE-EMS and C-QUATRE, which obtained a minimum objective function value of 0.0255 and an average runtime improvement of 98.8%. The optimized parameters of the proposed method yielded the Sum of Squared Errors (SSE) as low as 0.00002 in some cases, which indicates better precision and stability. Moreover, the voltage–current (V–I) and power–voltage (P–V) characteristics predicted by Di-DE were within 1% error relative to the experimental data for all tested PEMFCs. The results of this work highlight the potential of the Di-DE algorithm to enable more sophisticated modelling and optimization of PEMFCs, which in turn will help to broaden the use of PEMFCs in clean energy applications.

## Introduction

The need for power, the gradually depleting fossil fuels, and increased greenhouse gas emissions have necessitated the search for new technologies that can effectively harness renewable energy sources. PEMFCs are considered to be one of the most promising energy conversion technologies, and there are many reasons for this. For instance, PEMFCs have high efficiency in converting chemical energy into electrical energy with efficiencies greater than conventional combustion engines. They work at low temperatures ranging from 60 to 80 °C, this means that they can be brought online quickly with minimal thermal loads placed on the system elements, which leads to increased longevity of the system. Also, PEMFCs are characterized by high power density, which make them ideal for use in areas where space and weight are of the essence like in automobiles and portable power sources. Firstly, PEMFCs generate water only as a product, which makes them environmentally friendly since they do not emit any polluting gases during their operation. However, the systematic and chaotic nature of PEMFCs is a major challenge in their modelling and parameter identification. Accurate determination of model parameters is important for enhancing system efficiency, enhancing system design, and formulating efficient control measures.

PEMFC works based on the electrochemical reaction where hydrogen and oxygen gases are transformed into electricity, heat, and water. At the anode, hydrogen gas is broken down into protons (hydrogen ions) through a catalytic process. The protons pass through the proton exchange membrane which is a solid polymer electrolyte that only allows proton transport while the electrons are made to move through an external circuit thus generating an electric current that can be used to produce power. At the cathode, oxygen molecules combine with the incoming protons. This electrochemical reaction is a delicate process that involves controlling the flow of reactant gases, humidity levels to ensure that the membrane remains wet, temperature control, and management of the produced water to avoid overflooding or drying of the membrane. These factors are essential for the efficient functioning and durability of the PEMFC. To model these phenomena accurately, several parameters that affect the performance of the PEMFC^1^ must be determined, including activation overpotentials, ohmic resistances and concentration losses. Due to the coupling and nonlinearity of these parameters, it is crucial to apply sophisticated optimization methods to identify these parameters and predict the PEMFC performance under different conditions.

Fuel cells are now widely employed in commercial, industrial, and residential applications as both prime and backup sources of power because of their reliability and high conversion efficiency^2^. These cells are categorized based on the type of electrolyte they utilize and their initialization times. For instance, a Proton Exchange Membrane (PEM) fuel cell can be brought up to power in just one second, whereas a Solid Oxide (SO) fuel cell takes ten minutes to do so. Usually, a simple fuel cell can generate an output voltage of between 0 and 1 for applications that need higher voltage, several cells are connected in series PEM fuel cell is the most common due to the fact that it has no emissions, works at normal temperatures and pressures, and has a high efficiency^3,4^. It is therefore advisable to design and test a fuel cell model prior to installation in order to ease the design process and testing of the fuel cell model^5^. The development of models that represent the characteristics of a fuel cell has been an active area of research for the past two decades which helps in the understanding of the internal processes of the cells. This is important because the behavior of fuel cells is based on the characteristics of the model and the parameter values of the model that are often not available from the manufacturer’s datasheet. This stresses the need to estimate these uncertain parameters using appropriate optimization methods.

Fuel cell models are generally categorized into three types: The decision-making approaches are analytical, empirical, and hybrid^6^. Regardless of the approach taken, the aim is to find the best estimate of the parameters and this can be done using conventional^7,8^ or meta-heuristic^9^ optimization techniques. Previous techniques rely on iterative procedures or other numerical methods that are constrained by the initial conditions of the model, the number of iterations, and the model complexity^6–9^. On the other hand, metaheuristic optimization algorithms take advantage of computational power of the hardware, which has led to their use. In recent years^10^ introduces new gas channel architectures that effectively reduce water flooding problem, which is a major concern in PEMFCs. Based on this research, it is possible to continue the discussion on how the enhanced serpentine structures enhance the management of water as well as the performance of the cell. This integration will enable us to demonstrate how our Di-DE algorithm may tune the parameters of models with complex channel architectures, such as the one we have presented in this work, thus increasing the real-world usefulness of our research. Another source^11^ provides information on new flow field configurations that improve water distribution through the use of variable area cross-sections. With this work included, we can focus on the effect of flow field structures on the performance of PEMFC. We will explain how our parameter estimation methodology can be applied to these new designs and how it can still effectively model and optimize PEMFCs as their architecture changes. This paper^12^ discusses a new approach to assessing the diffusion of reactants and the impact on PEMFC performance. Thus, we can enhance our argument on the significance of correctly assessing mass transport parameters. This will demonstrate how our Di-DE algorithm tackles problems of diffusion rate estimations and, thus, helps to design better fuel cells. Another study^13^ investigates the effects of variable section flow fields and mass transfer and the under-convection flow to improve the PEMFC performance. Thus, incorporating this research enables us to explain the contemporary developments in the flow field designs that enhance the mass transfer. We will discuss these observations in the context of our optimization framework and show how our algorithm can handle such design enhancements in parameter estimation.

Currently, metaheuristic algorithms are extensively employed for an efficient estimation of the parameters in various engineering applications like electric machines and optimal power flow in power systems^14^. For instance, several meta heuristic algorithms have been used in fuel cells (FCs) that include grey wolf optimization-cuckoo search algorithm (GWO-CS)^6^, grey wolf optimization (GWO)^6^, chaotic mayfly optimization algorithm (CMOA)^6^, and other algorithms such as neural network algorithm (NNA)^15^, firefly optimization algorithm (FOA), and imperialist-competitive algorithm (ICA). Other algorithms like shuffled frog-leaping algorithm (SFLA)^16^, marine predator algorithm (MPA)^17^, and others, such as the Hunger Games Search Algorithm^18^, Manta Rays Foraging Optimizer^19^, Whale Optimization Algorithm^20^, Grasshopper Optimizer^21^, Seeker Optimization Algorithm^22^,^23^, Bird Mating Optimizer^24^, Grey Wolf Optimizer^25^, Flower Pollination Algorithm^26^, Atom Search Optimizer^27^, Hybrid Adaptive Differential Evolution Algorithm^28^, Hybrid Artificial Bee Colony Algorithm^29^, Teaching–Learning-Based Optimization (TLBO)^30^, Biogeography-Based Optimization (BBO)^31^, and Backtracking-Search Algorithm^32^, Lightning search algorithm^33^, QUATRE-EMS^34^ have also been employed to enhance the modeling of power system.

Fuel cells are a key enabling technology for sustainable energy systems, and have the potential to decarbonize transportation and power generation. Proton Exchange Membrane Fuel Cells (PEMFCs) are among the various types, which are attractive for their high efficiency, compact design and low environmental impact, and are therefore suitable for use in hybrid electric vehicles and distributed energy systems^35^. Recently, there have been major inroads in the available modeling and optimization techniques that have increased PEMFC systems performance and reliability. For example, algorithms based on multiple learning neural network have shown high accuracy in parameter estimation of PEMFC models and improved their predictive capabilities^36^. Innovative energy management strategies for multi-stack fuel cell systems have been proposed to tackle the challenges of high power applications and to provide more robust and efficient architectures^37^. Furthermore, PEMFC degradation prediction has been performed using optimization techniques such as whale optimization algorithms to increase their operational lifespan^38^. Dynamic semi empirical models for fault diagnosis and prognostics^39^, improved ambient condition based dynamic models for hydration state detection^40^ and advanced dynamic models for fault tolerant operation^41^ are some of the early research that has helped in the development of PEMFC models. Additionally, we report on the use of parameter identification methods based on optimization techniques such as particle swarm optimization, which holds promise for improving model accuracy and performance^42^. Building on these foundations, this study seeks to fill critical gaps in PEMFC modeling and optimization.

The extensive application of these methods points towards the continuous work being done in the enhancement of PEMFC modeling through various optimization strategies. However, the field of meta-heuristic optimization is still growing to this date, which offers potential for the design of new algorithms. This is even more applicable when considering the no-free-lunch theorem, which indicates that it is helpful to consider a range of optimization techniques when addressing diverse engineering concerns, since one approach may not be optimal for all problems^43^. One of the main benefits of metaheuristic algorithms is their ability to work regardless of the initial state and quickly search for solutions. However, their major drawback is that they may converge sub-optimally if fewer iterations are used. Therefore, there is a shift towards developing metaheuristic algorithms that are a combination of the two, to achieve the best results with the least number of iterations.

In conclusion, despite the fact that the number of metaheuristic algorithms utilized in PEMFC modeling is DE is a very simple yet efficient metaheuristic method to solve optimization problems. It has gained much popularity in recent years because it is easy to use and yields good results. Nevertheless, there are some factors that can affect the effectiveness of the DE algorithm, including mutation strategy and parameter control in the trial vector generation. These elements are usually plagued by problems like early solutions at local optima and interdependencies of control parameters. To overcome these challenges, the Depth Information-Based Differential Evolution (Di-DE) algorithm was proposed in this paper. This improvement involves the integration of an external archive that uses depth information in the mutation strategy to enhance the understanding of the optimization landscape. Moreover, Di-DE employs a specific grouping mechanism that allows for the updating of parameters in isolation, thus eliminating the problem of incorrect parameter correlations. The improvements in Di-DE have demonstrated better results than other optimization techniques. The experimental results based on CEC2013^44^ and CEC2017 benchmark sets have shown that this algorithm performs comparable to the PSO variants, QUATRE variants, and other DE variants. The reason for using Di-DE with Adaptive Parameter Control is to improve the modelling of Proton Exchange Membrane Fuel Cells (PEMFCs). This study highlights several key contributions:

1. Utilization of Di-DE for optimal parameter selection in various PEMFC models such as BCS500W^45^, NedStackPS6^46^, S12^46^, Standard250W^47^, H12^48^, and HORIZON^48^. This algorithm variant enhances convergence speed and effectively avoids local optima, leading to more accurate estimations of unknown parameters.
2. A comparative analysis was conducted between Di-DE and other DE variants like iwPSO^49^, CLPSO^50^, DNLPSO^51^, SLPSO^52^, SaDE^53^, JADE^54^, SHADE^55^, QUATRE^56^, LSA^33^, QUATRE-EMS^34^ and C-QUATRE^57^, assessing their efficacy in optimizing PEMFC parameters.
3. The robustness and consistency of Di-DE were evaluated by comparing calculated I–V and P–V characteristic curves with actual measured data.
4. Experimental results confirmed that Di-DE outperforms all other compared techniques, delivering significantly better and distinct outcomes for the PEMFC parameter optimization challenge.

The structure of the remainder of the paper is as follows: Section "Mathematical PEMFC stack modeling" delves into the mathematical formulation of PEMFCs and the objective function. Section "Novel DI-DE algorithm" provides an overview of the Di-DE algorithm. Section "Experimental analysis" details the simulations and the results obtained. Finally, Sect. 5 concludes the paper with a summary of the findings.

## Mathematical PEMFC stack modeling

The steady-state behaviour of the Proton Exchange Membrane Fuel Cell (PEMFC) is captured using an electrochemical model developed by Amphlett et al. as detailed in references^58,59^. In this model, the output voltage of the PEMFC (denoted as ${V}_{\text{FC}}$) is calculated as the sum of the cell reversible voltage (${E}_{\text{Nernst}}$) and three types of voltage losses: activation (${V}_{\text{Act}}$), ohmic (${V}_{\text{Ohmic}}$), and concentration (${V}_{\text{Con}}$). The model applies to a series connection of multiple cells (${N}_{\text{cell}}$) and assumes uniform behaviour across all cells. The overall expression for this electrochemical model is presented as follows:

1 $${V}_{\text{FC}}={N}_{\text{cell}}\left({E}_{\text{Nernst}}-{V}_{\text{Act}}-{V}_{\text{Ohmic}}-{V}_{\text{Con}}\right)$$

The Nernst equation, which calculates the thermodynamic potential, is derived from^21,48^ as follows:

2 $$\begin{aligned}{E}_{\text{Nernst}}&=1.229-0.85\times {10}^{-3}\left({T}_{\text{stack}}-298.15\right)+4.3085\\ &\quad\times {10}^{-5}{T}_{\text{stack}}\left(\text{ln}({P}_{{\text{H}}_{2}})+0.5\text{ln}({P}_{{\text{O}}_{2}})\right)\end{aligned}$$

where ${T}_{\text{stack}}$ is the stack temperature (K), ${P}_{{\text{H}}_{2}}$ is the hydrogen partial pressure at the anode (atm), and ${P}_{{\text{O}}_{2}}$ is the oxygen partial pressure at the cathode (atm). The partial pressures of reactants in the inlet flow channels of a PEMFC are influenced by the humidification levels of the inlet streams and the consumption rates of oxygen and hydrogen^58,59^.

In scenarios where air and hydrogen are the reactants, the partial pressure of oxygen (${P}_{{\text{O}}_{2}}$) can be determined as follows^60,61^:

3 $$\begin{aligned}{P}_{{\text{O}}_{2}}={P}_{\text{C}}-(R{H}_{\text{C}}{P}_{{\text{H}}_{2}{\text{O}}}^{\text{sat}})-\frac{0.79}{0.21}{P}_{{\text{O}}_{2}}exp\left(0.291\frac{{I}_{\text{FC}}}{A}\left({T}_{\text{stack}}^{0.832}\right)\right) {\text{(air} \, {\text{and}} \, {\text{H}}}_{2})\end{aligned}$$

If oxygen and hydrogen are the reactants, ${P}_{{\text{O}}_{2}}$ is calculated as follows^62,63^:

4 $$\begin{aligned}{P}_{{\text{O}}_{2}}=R{H}_{\text{C}}{P}_{{\text{H}}_{2}{\text{O}}}^{\text{sat}}{\left[\left(\text{exp}\left(4.192\frac{{I}_{\text{FC}}}{A}\left({T}_{\text{stack}}^{1.334}\right)\right)\right)\times \frac{R{H}_{\text{C}}{P}_{{\text{H}}_{2}{\text{O}}}^{\text{sat}}}{{P}_{\text{C}}}\right]}^{-1}\end{aligned}$$

In both cases, ${P}_{{\text{H}}_{2}}$ is given by^64,65^:

5 $$\begin{aligned}{P}_{{\text{H}}_{2}}=0.5R{H}_{\text{a}}{P}_{{\text{H}}_{2}{\text{O}}}^{\text{sat}}\left[{\left(\text{exp}\left(1.635\frac{{I}_{\text{FC}}}{A}\left({T}_{\text{stack}}^{1.334}\right)\right)\times \frac{R{H}_{\text{C}}{P}_{{\text{H}}_{2}{\text{O}}}^{\text{sat}}}{{P}_{\text{a}}}\right)}^{-1}-1\right]\end{aligned}$$

Here, $R{H}_{\text{C}}$ and $R{H}_{\text{a}}$ represent the relative humidity of vapor in the electrodes, ${P}_{\text{C}}$ and ${P}_{\text{a}}$ are the cathode and anode inlet partial pressures (atm), ${I}_{\text{FC}}$ is the PEMFC operating current (A), $A$ is the active area of the membrane (cm^2^), and ${P}_{{\text{H}}_{2}{\text{O}}}^{\text{sat}}$ is the saturation water vapor pressure (atm). The saturation vapor pressure at the fuel cell operating temperature can be defined as^60,66^:

6 $$\begin{aligned}{\text{log}}_{10}({P}_{{\text{H}}_{2}{\text{O}}}^{\text{sat}})&=2.95\times {10}^{-2}({T}_{\text{stack}}-273.15)-9.18\times {10}^{-5}({T}_{\text{stack}}-273.15{)}^{2}\\ &\quad+1.44\times {10}^{-7}({T}_{\text{stack}}-273.15{)}^{3}-2.18\end{aligned}$$

The activation loss is the overpotential required to activate the electrodes. This loss dominates in low current density regions and is calculated as:

7 $$\begin{aligned}{V}_{\text{Act}}=-\left[{\xi }_{1}+{\xi }_{2}{T}_{\text{stack}}+{\xi }_{3}{T}_{\text{stack}}\text{ln}({C}_{{\text{O}}_{2}})+{\xi }_{4}{T}_{\text{stack}}\text{ln}({I}_{\text{FC}})\right]\end{aligned}$$

where ${C}_{{\text{O}}_{2}}=\left(\frac{{P}_{{\text{O}}_{2}}}{5.08}\right)\times {10}^{6}\text{exp}\left(-\frac{498}{{T}_{\text{stack}}}\right)$ represents the oxygen concentration (mol/cm^3^), and ${\xi }_{k}$ (where $k=1\dots 4$) are semi-empirical coefficients derived from theoretical equations integrating kinetic, thermodynamic, and electrochemical principles^67^. These parameters are determined by solving the Butler-Volmer equation, which considers factors like the transfer coefficient, exchange current density, universal gas constant, Faraday constant, and the number of electrons involved in the reactions.

The ohmic voltage drop arises from resistance to electron transfer through the collecting plates and carbon electrodes and proton transfer through the solid membrane. It is quantified using the following general formula^68,69^:

8 $${V}_{\text{Ohmic}}={I}_{\text{FC}}\left({R}_{m}+{R}_{\text{C}}\right)$$

The membrane resistance ${R}_{m}$ is expressed as: ${R}_{m}=\frac{{\rho }_{m}l}{A}$ where ${\rho }_{m}$ is the membrane resistivity (Ω.cm), $l$ is the membrane thickness, and $A$ is the active area. The resistivity ${\rho }_{m}$ is a function of the water content in the membrane and is defined as: ${\rho }_{m}=\frac{181.6[1+0.03J+0.062({T}_{\text{stack}}/303{)}^{2}{J}^{2.5}]}{[\lambda -0.643-3J]\text{exp}(4.18({T}_{\text{stack}}-303)/{T}_{\text{stack}})}$ In this expression, $J$ represents the current density (A/cm^2^), and $\lambda$ is an adjustable parameter dependent on the membrane’s water content, which can range from 10 to 23 based on relative humidity and stoichiometry. Unlike earlier assumptions, ${R}_{\text{C}}$ is not a constant but can vary based on electrode preparation, manufacturing quality, and membrane conditions^70,71^.

Concentration losses result from mass transport limitations, reducing the reactant concentrations at the electrodes. The concentration voltage drop is given by:

9 $${V}_{\text{Con}}=-\beta ln\left(\frac{{J}_{\text{max}}-J}{{J}_{\text{max}}}\right)$$

where $\beta$ is a parametric coefficient (V) depending on the cell and its operational state^71^, ${J}_{\text{max}}$ is the maximum current density, and $J$ is the actual current density (A/cm^2^). ${J}_{\text{max}}$ represents the point where fuel delivery becomes the limiting factor for current production.

Our goal was to determine seven crucial nonlinear parameters of the Proton Exchange Membrane Fuel Cell (PEMFC) mathematical model with high accuracy. These parameters are important because they provide the detailed information on the electrochemical processes and losses that occur in the fuel cell and, therefore, directly impact the fuel cell efficiency. The seven parameters are: ${{\varvec{\xi}}}_{1}$ parameter is contained in the activation overpotential equation and is associated with the electrode kinetics. It effects the reaction kinetics of the electrochemical reactions occurring at the electrodes, thus affecting the activation loss of the fuel cell. ${{\varvec{\xi}}}_{2}$ describes the influence of temperature on the activation overpotential. It shows the dependency of the reaction kinetics and the associated voltage losses on the operating temperature of the PEMFC. The ${{\varvec{\xi}}}_{3}$ parameter involves the concentration of the reactants and their effects on the activation overpotential. It depicts the effect of the hydrogen and oxygen partial pressures on the cell voltage. ${{\varvec{\xi}}}_{4}$ is related to the logarithmic relation between the activation overpotential and the current density. It determines how the voltage loss increases with higher current densities due to activation polarization. ${\varvec{\lambda}}$ Lambda represents the degree of hydration in the polymer electrolyte membrane. It affects the proton conductivity of the membrane, influencing the ohmic losses in the fuel cell. ${{\varvec{R}}}_{{\varvec{c}}}$ accounts for the resistive losses due to electron flow through the cell components, including the gas diffusion layers and bipolar plates. It contributes to the overall ohmic losses in the PEMFC. The parameter ***B*** is involved in modeling the concentration overpotential, which arises from mass transport limitations at high current densities. It reflects the voltage loss due to the depletion of reactants at the electrode surfaces.

### Fitness function definition

Typically, the optimization problem is formulated by defining a fitness function that serves as the minimization objective, with decision variables identified as the parameters to be estimated. The search space is delineated by the upper and lower bounds of each decision variable. Optimization algorithms use this fitness function to guide the population toward improved solutions. The primary goal of this fitness function is to derive the steady-state model parameters by minimizing the sum of squared errors (SSE) between the observed output voltage of each PEMFC stack and the voltage predicted by the model. The rationale for using this fitness function lies in its prevalence in the field, which allows for a direct comparison of the results from this study with those obtained from other optimizers discussed in existing publications. The formulation of this fitness function is as follows:

10 $$\left\{\begin{array}{l}\mathop{{\bf min}}\limits_{\text{(SSE)}}\sum_{i=1}^{N}{({V}_{FC,meas}(i)-{V}_{FC,est}(i))}^{2}\\ {\xi }_{k,min}\le {\xi }_{k}\le {\xi }_{k,max}\left({\text{k}}=1\dots 4\right)\\ {R}_{C,min}\le {R}_{C}\le {R}_{C,\text{max}}\\ {\lambda }_{min}\le \lambda \le {\lambda }_{max}\\ {\beta }_{min}\le \beta \le {\beta }_{max}\end{array}\right.$$

where, ${V}_{\text{FC,meas}}$ represents the measured output voltage, ${V}_{\text{FC,est}}$ is the output voltage estimated by the model, and $N$ denotes the number of sample data points. The accuracy of the estimated parameter values is evaluated by simulating the described PEMFC models using MATLAB software. It is important to emphasize that the choice of appropriate initial parameter values plays a crucial role in the quality of the estimation process. In this study, the fitness function is subject to practical inequality constraints, defined by the upper and lower limits of the parameters.

## Novel DI-DE algorithm

In this section, we provide an in-depth examination of the novel Depth Information-Based Differential Evolution (Di-DE) algorithm^44^. The explanation of the algorithm is structured into two main segments. Initially, the depth information-based mutation strategy is described. Following that, the adaptation schemes for the control parameters are detailed.

### Depth information based mutation strategy

The mutation strategy plays a crucial role in the Differential Evolution (DE) algorithm as it is responsible for generating the mutant vector for each individual, thereby defining the search range for each participant. Various mutation strategies exhibit distinct characteristics and, as a result, achieve varying performance levels across different objectives. In this context, we introduce a mutation strategy based on depth information for numerical optimization. This depth information is derived from historical individuals preserved in an external archive. Typically, the historical positions of individuals during the evolutionary process provide insights into the landscape of the objectives. Consequently, the relationships among these historical solutions can be analyzed and utilized to guide the evolutionary process. This approach helps navigate the population away from potential local optima while enhancing convergence speed. The integration of this archival data and the depth information it yields is incorporated into the mutation strategy, which influences the search direction at each stage in DE. The specifics of this mutation strategy are outlined in Eq. (11):

11 $${V}_{i,G}={X}_{i,G}+F\cdot ({X}_{{\text{best}},G}^{p}-{X}_{i,G})+F\cdot ({X}_{{r}_{1},G}-{\hat{X}}_{{r}_{2},G})$$

In this formula, ${X}_{{\text{best}},G}^{p}$ represents an individual chosen from the top $100p\text{\%}$ of the current population $P$; ${X}_{i,G}$ is the target vector; ${X}_{{r}_{1},G}$ and ${\hat{X}}_{{r}_{2},G}$ are individuals randomly selected from the current population $P$ and the combined set $P\cup A$, respectively, where $A$ is the external archive that stores historical solutions. Additionally, the size of $A$ is determined by ${r}_{\text{arc}}\cdot NP$. This mutation strategy bears similarities to that used in JADE, particularly concerning ${\hat{X}}_{{r}_{2},G}$. A more detailed examination of the role of the external archive is discussed in the experiment section.

### Parameter control

In the Di-DE algorithm, individuals are grouped into $K$ categories using stochastic universal selection^72^ during the initialization phase, with the selection probability for each group set to $P(j)=\frac{1}{K},j=\{1,2,\ldots ,K\}$. The scale factor $F$ and crossover rate $CR$ for each individual are distributed according to a Cauchy distribution and a Gaussian distribution, respectively, denoted as $F\sim C({x}_{F},{\gamma }_{F})$ and $CR\sim N({\mu }_{CR},{\sigma }_{CR})$. If the ${i}^{th}$ individual belongs to the ${j}^{th}$ group, its associated $F$ value is denoted as ${F}_{ji}$. Similarly, $C{R}_{ji}$ represents the crossover rate of the ${i}^{th}$ individual in the ${j}^{th}$ group. The initial values for ${x}_{F}$ and ${\mu }_{CR}$ in each group are set to 0.5:${x}_{{F}_{j}}={x}_{F}=0.5, {\mu }_{C{R}_{j}}={\mu }_{CR}=0.5, j\in \{\text{1,2},\dots ,K\}$ and the values of ${\gamma }_{F}$ and ${\sigma }_{CR}$ are both set to 0.1: ${\gamma }_{{F}_{j}}={\gamma }_{F}=0.1, {\sigma }_{C{R}_{j}}={\sigma }_{CR}=0.1, j\in \{1,2,\ldots ,K\}$ Following initialization, the Di-DE algorithm produces trial vectors based on its mutation strategy and crossover operation. If a trial vector improves upon its corresponding target vector, it is labeled as the "s" (successful) individual; otherwise, it is labeled as the "f" (failed) individual. Subsequently, the selection probability $P(j)$ is updated according to Eq. (12):

12 $$\left\{\begin{array}{l}ns=\sum_{j=1}^{K} n{s}_{j}\\ {r}_{j}=\left\{\begin{array}{ll}\frac{n{s}_{j}^{2}}{ns\cdot \left(n{s}_{j}+n{f}_{j}\right)},& \, {\text{if}} \, n{s}_{j}>0\\ \epsilon ,{r}_{j}& \, {\text{otherwise}} \, \end{array}\right.\\ P(j)=\frac{{r}_{j}}{\sum_{j=1}^{K} \left({r}_{j}\right)}\end{array}\right.$$

In this model, $n{s}_{j}\left(n{f}_{j}\right)$ represents the number of ' ('f') individuals within the ${j}^{\text{th}}$ group, where $ns$ is the aggregate count of ' individuals across the entire population. Here, $P(j)$ refers to the selection probability for the ${j}^{\text{th}}$ group. Upon calculating the selection probability for each group, the parameters ${x}_{{F}_{j}}$ and ${\mu }_{C{R}_{j}}$ are updated in accordance with Eqs. (13) and (14), respectively:

13 $$\left\{\begin{array}{l}\Delta {f}_{i}=f\left({U}_{i,G}\right)-f\left({X}_{i,G}\right)\\ {w}_{{F}_{ji}}=\frac{\Delta {f}_{i}}{\sum_{{F}_{ji}\in {S}_{{F}_{j}}}\Delta {f}_{i}}\\ {\text{mean}}_{WL}\left({S}_{{F}_{j}}\right)=\frac{\sum_{{F}_{ji}\in {S}_{{F}_{j}}} {w}_{{F}_{j,i}}\cdot {F}_{ji}^{2}}{\sum_{{F}_{ji}\in {F}_{j}} {w}_{{F}_{ji}}\cdot {F}_{ji}}\\ {x}_{{F}_{j}}=c(j)\cdot {x}_{{F}_{j}}+(1-c(j))\cdot { \, {\text{mean}} \, }_{WL}\left({S}_{{F}_{j}}\right)\end{array}\right.$$

14 $$\left\{\begin{array}{l}\Delta {f}_{i}=f\left({U}_{i,G}\right)-f\left({X}_{i,G}\right)\\ {w}_{C{R}_{ji}}=\frac{\Delta {f}_{i}}{\sum_{C{R}_{ji}\in {S}_{C{R}_{j}}}\Delta {f}_{i}}\\ {\text{mean}}_{WL}\left({S}_{C{R}_{j}}\right)=\frac{\sum_{C{R}_{ji}\in {S}_{C{R}_{j}}} {w}_{C{R}_{ji}}\cdot C{R}_{ji}^{2}}{\sum_{C{R}_{ji}\in C{R}_{j}} {w}_{C{R}_{ji}}\cdot C{R}_{ji}}\\ {\mu }_{C{R}_{j}}=c(j)\cdot {\mu }_{C{R}_{j}}+(1-c(j))\cdot { \, {\text{mean}} \, }_{WL}\left({S}_{C{R}_{j}}\right)\end{array}\right.$$

where $c(j)$ satisfies Eq. (15):

15 $$c(j)=\left\{\begin{array}{ll}\frac{n{s}_{j}}{n{s}_{j}+n{f}_{j}},& \, {\text{if}} \, n{s}_{j}+n{f}_{j}>0\\ 0.& \, {\text{otherwise}}\end{array}\right.$$

**Fig. 1:**
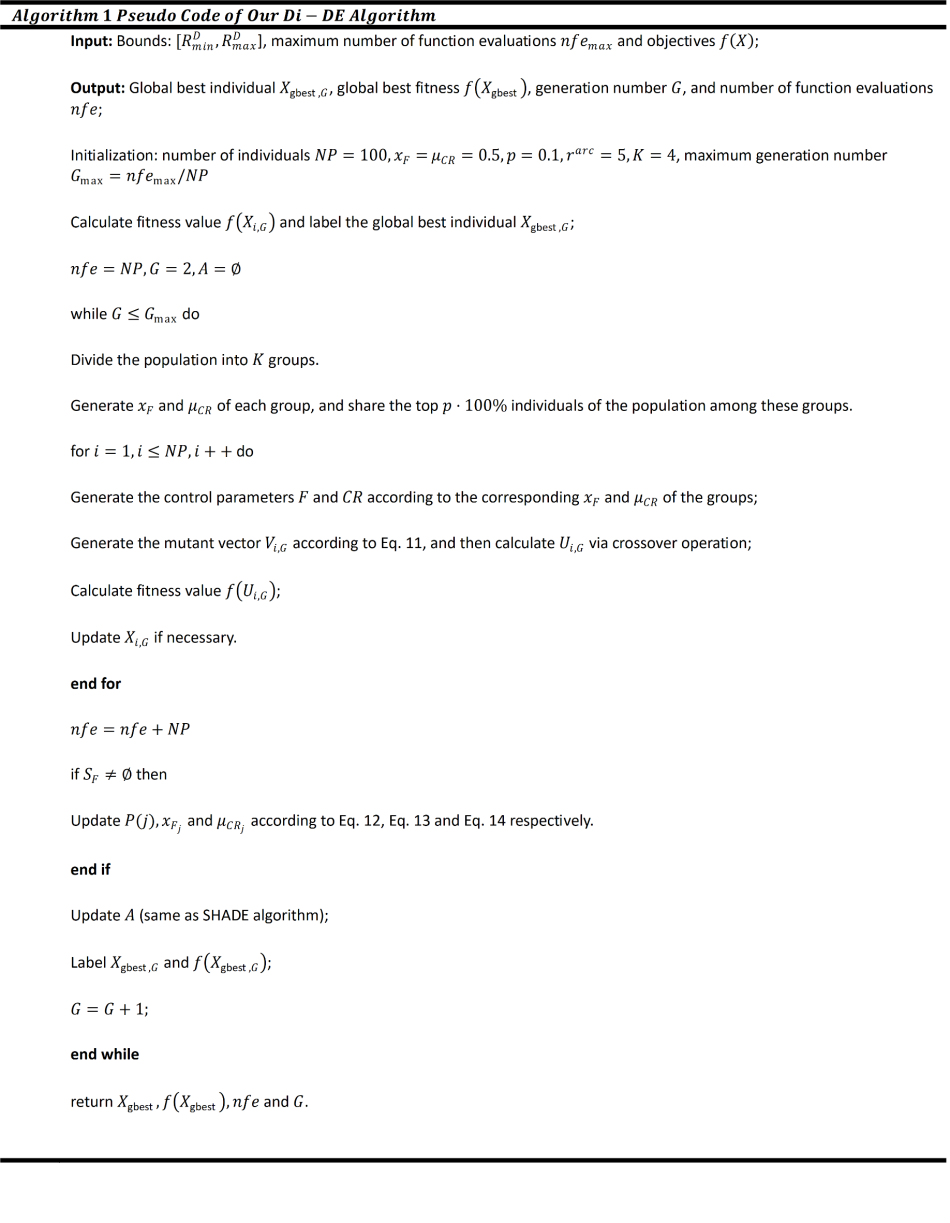
Pseudo code of Di-DE algorithm.

For each generation, the values of ${x}_{F}$ and ${\mu }_{CR}$ for all $K$ groups are updated, enhancing the performance of the innovative Di-DE algorithm. The pseudocode for the Di-DE algorithm is outlined in Fig. 1.

## Experimental analysis

In addition to the evolution matrix, the DI-DE algorithm introduces a novel selection operator. This operator incorporates some suboptimal solutions during the evolution process, akin to selecting the top percentage of individuals in the population as part of the mutation strategy. This selection mechanism enhances the algorithm ability to escape local optima during its evolutionary course. To validate the algorithm, a comprehensive test suite of 12 benchmarks was utilized, and the results underscore the enhanced performance of Differential Evaluation (DE) algorithm namely, Depth Information-Based Differential Evolution (Di-DE) algorithm over previous leading Differential Evolution (DE) variants, as detailed in^44^. This superiority of the Di-DE algorithm is specifically applied and confirmed in the optimization of PEMFC parameters. For validation, the results using the QUATRE algorithm were benchmarked against nine different DE variants, including iwPSO^49^, CLPSO^50^, DNLPSO^51^, SLPSO^52^, SaDE^53^, JADE^54^, SHADE^55^, QUATRE^56^, C-QUATRE^57^, LSA^33^, QUATRE-EMS^34^ with default parameter settings are given in Table 1. All algorithms compared were set to their recommended to estimate the parameter of a PEMFC fuel cell (BCS500W^45^, NedStackPS6^46^, S12^46^, Standard250W^47^, H12^48^, and HORIZON^48^) presented in Table 2. All the experiments are carried out on Matlab 2021a of a PC with Windows Server 2019 operating system CPU i7-11700 k@3.6 GHz, maximum iterations 500, number of run 30 and population size 40. The maximum number of iterations was set to 500 based on preliminary convergence studies. These studies showed that the Di-DE algorithm consistently reached convergence before the 500-iteration mark across all PEMFC models tested. This iteration limit balances computational efficiency with solution accuracy, ensuring that the algorithm has sufficient opportunity to explore the search space and refine solutions without incurring unnecessary computational costs. The selection aligns with common practices in the literature and was deemed appropriate given the algorithm’s rapid convergence characteristics demonstrated in our experiments.

**Table 1:** Default parameter settings of the DE algorithms in the comparison.

Algorithm	Parameters initial settings
iwPSO^49^	${c}_{1}={c}_{2}=2.0,w\in [\text{0.9,0.4}]$, vel $=rnd$
CLPSO^50^	$w\in [\text{0.9,0.3}],cc=1.49455,Pc\in [\text{0,0.5}]$, stay_num $=7$, vmax $=0.2R$
DNLPSO^51^	${c}_{1}={c}_{2}=1.49445,w\in [\text{0.9,0.4}],Pc\in [\text{0.45,0.05}],m=3,g=5$
SLPSO^52^	$M=100,{c}_{3}=0.005,PL\in [\text{0,1}]$
SaDE^53^	$NP=50,F\sim N\left({\mu }_{F},0.3\right),{\mu }_{F}=0.5,CR\sim N\left({\mu }_{CR},0.1\right),{\mu }_{CR}=0.5,LP=50$
JADE^54^	$NP=100,F\sim C\left({\mu }_{F},0.1\right),{\mu }_{F}=0.5,CR\sim N\left({\mu }_{CR},0.1\right),{\mu }_{CR}=0.5,p=0.05,c=0.1$
SHADE^55^	$NP=100,F\sim C\left({\mu }_{F},0.1\right),{\mu }_{F}=0.5,CR\sim N\left({\mu }_{CR},0.1\right),{\mu }_{CR}=0.5,p=0.2,\text{H}=100$
QUATRE^56^	$NP=100,F=0.7$
C-QUATRE^57^	$NP=100,F=0.7$
Di-DE^44^	$NP=10\cdot D,F\sim C\left({\mu }_{F},0.1\right),{\mu }_{F}=0.5,CR\sim N\left({\mu }_{CR},0.1\right),{\mu }_{CR}=0.5,p=0.1,{r}^{arc}=5,K=4$

**Table 2:** Characteristics of twelves PEMFCs used in this work.

S. no.	PEMFC type	Power (W)	Ncells (no)	A (cm^2^)	L (um)	T (K)	Jmax (mA/cm^2^)	PH_2_ (bar)	PO_2_ (bar)
FC1	BCS 500 W	500	32	64	178	333	469	1.0	0.2095
FC2	NetStack PS6	6000	65	240	178	343	1125	1.0	1.0
FC3	SR-12	500	48	62.5	25	323	672	1.47628	0.2095
FC4	H-12-1	12	13	8.1	25	323	246.9	0.4935	1.0
FC 5	Ballard Mark V	5000	35	232	178	343	1500	1.0	1.0
FC 6	STD-1	250	24	27	127	343	860	1.0	1.0
FC 7	Horizon	500	36	52	25	338	446	0.55	1.0
FC8	STD-2	250	24	27	127	343	860	1.5	1.5
FC9	STD-3	250	24	27	127	343	860	2.5	3.0
FC10	STD-4	250	24	27	127	353	860	2.5	3.0
FC11	H-12-2	12	13	8.1	25	302	246.9	0.4	1.0
FC12	H-12-3	13	13	8.1	25	312	246.9	0.5	1.0

### PEMFC FC1

**Fig. 2:**
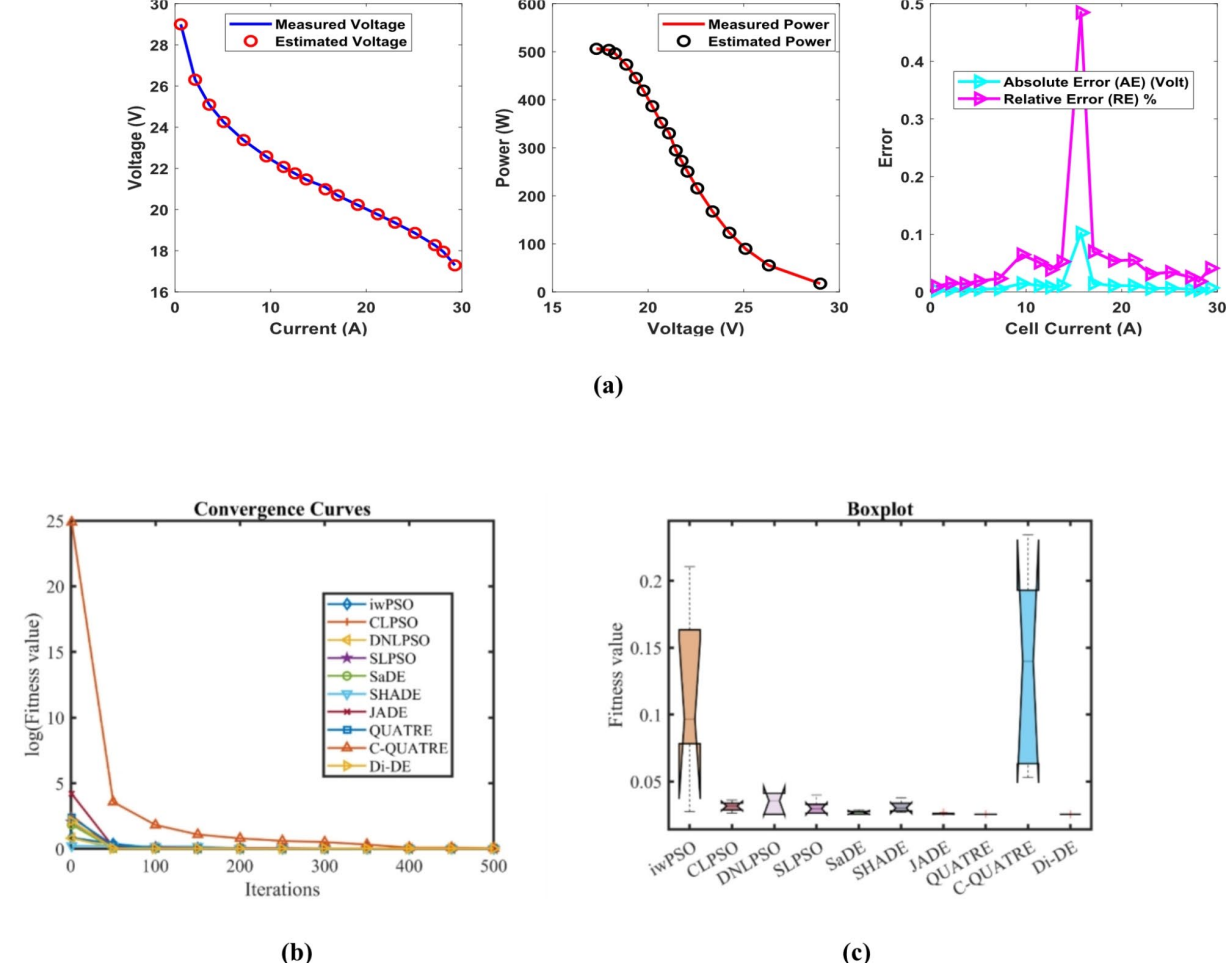
FD-DE Algorithm Characteristic curves of FC1; (**a**) V-I, P–V, and Error curve, (**b**) Convergence Curve, (**c**) Box-Plot.

In Table 3 the Di-DE algorithm stands out for its exceptional stability, precision, and efficiency. The minimum, maximum, and mean values for Di-DE are remarkably consistent at 0.0254927, 0.0254976, and 0.0254937, respectively, indicating negligible fluctuation and extraordinary consistency across multiple test scenarios. This minimal variation is further underscored by Di-DE standard deviation of just 2.21E-06, which is significantly lower than its closest competitor, QUATRE, with a standard deviation of 5.05E-05. This stark contrast highlights Di-DE superior predictability and reliability in delivering stable results. In terms of runtime efficiency, Di-DE is unparalleled, recording a minimal 0.1232347s, notably faster than other algorithms such as SaDE, which has a runtime of 10.627793s, demonstrating an efficiency improvement of approximately 98.84%. Compared to other algorithms, Di-DE consistently shows superior performance. For instance, iwPSO, with a minimum value of 0.0275149, has a much higher mean of 0.1154848 and a standard deviation of 0.0681369, indicating significant variability and less consistency. CLPSO and DNLPSO, while better than iwPSO, still exhibit higher mean values of 0.0313693 and 0.0337062, respectively, and higher standard deviations of 0.0036649 and 0.0078276, showing more variability. SLPSO, with a mean of 0.0305522 and standard deviation of 0.0055962, also falls short of Di-DE precision. SaDE, despite having a relatively low mean value of 0.0267998 and a standard deviation of 0.0012763, is much less efficient due to its significantly higher runtime. SHADE, with a mean of 0.0311506 and a standard deviation of 0.0042825, shows more variability and less stability. JADE, while close to Di-DE with a mean of 0.0259319 and a very low standard deviation of 0.0005081, still exhibits slightly higher variability. QUATRE, despite its competitive mean of 0.0255291, has a higher standard deviation compared to Di-DE, indicating less consistency. C-QUATRE performs poorly with a high mean of 0.1346057 and a significant standard deviation of 0.0762099, indicating high variability and inconsistency. This detailed analysis reinforces in Tables 3, 4 and Fig. 2 shows Di-DE prowess in achieving optimal results with minimal computational overhead, superior stability, and efficiency compared to other algorithms, making it particularly valuable in applications where precision, reliability, and efficiency are paramount.

**Table 3:** Optimized parameters and optimal function value for FC1.

Algorithm	iwPSO	CLPSO	DNLPSO	SLPSO	SaDE	SHADE	JADE	QUATRE	C-QUATRE	Di-DE
${\xi }_{1}$	-0.9873122	-0.8532228	-1.19969	-0.9515123	-1.113918	-0.8632478	-0.8730994	-1.1775119	-0.8990113	-1.1472021
${\xi }_{2}$	0.0034775	0.002318	0.0041839	0.0030656	0.0036387	0.0030058	0.0030698	0.0035848	0.0032187	0.0040262
${\xi }_{3}$	9.359E-05	4.484E-05	0.000098	7.397E-05	7.948E-05	8.731E-05	8.94E-05	6.373E-05	9.388E-05	9.799E-05
${\xi }_{4}$	-0.0001919	-0.0001944	-0.000193	-0.0001938	-0.0001929	-0.0001926	-0.0001928	-0.000193	-0.0001852	-0.000193
$\lambda$	21.491265	22.127812	20.877245	22.567593	21.824809	21.368614	21.628903	20.929105	17.500522	20.877243
${R}_{c}$	0.0002614	0.000103	0.0001	0.0001963	0.0001886	0.000118	0.0001776	0.0001003	0.0002992	0.0001
*B*	0.0153331	0.0167648	0.0161261	0.0163309	0.0161754	0.0162822	0.0161769	0.0161601	0.0136	0.0161261
*Min*	0.0275149	0.0264193	0.0254927	0.0261257	0.0255704	0.0269552	0.0256314	0.0254974	0.0530034	0.0254927
*Max*	0.2105302	0.0361364	0.0410172	0.0399463	0.0286518	0.0378559	0.0268311	0.0256179	0.234402	0.0254976
*Mean*	0.1154848	0.0313693	0.0337062	0.0305522	0.0267998	0.0311506	0.0259319	0.0255291	0.1346057	0.0254937
*Std*	0.0681369	0.0036649	0.0078276	0.0055962	0.0012763	0.0042825	0.0005081	5.049E-05	0.0762099	2.205E-06
*RT*	4.727	3.8201111	2.8949521	3.0213271	10.627793	3.4800649	3.4996465	4.1785178	9.4803796	0.1232347
*FR*	8.8	6.8	5.4	5.8	4.8	6.6	3.8	2.2	9.4	1.4

**Table 4:** Performance metrics of FD-DE Algorithm for FC1.

S. no.	*I*_*exp*_ (A)	*V*_*exp*_ (V)	*V*_*est*_ (V)	*P*_*exp*_ (W)	*P*_*est*_ (W)	*AE*_*v*_ (A)	*RE %*	*MBE*
1	0.6	29	28.9972233	17.4	17.398334	0.00277671	0.00957487	4.2834E-07
2	2.1	26.31	26.3059371	55.251	55.2424678	0.00406294	0.01544257	9.17083E-07
3	3.58	25.09	25.0935552	89.8222	89.8349278	0.00355524	0.01416994	7.02206E-07
4	5.08	24.25	24.2546203	123.19	123.213471	0.0046203	0.01905277	1.18595E-06
5	7.17	23.37	23.3754161	167.5629	167.601733	0.00541606	0.02317526	1.62965E-06
6	9.55	22.57	22.584615	215.5435	215.683073	0.01461499	0.06475407	1.18666E-05
7	11.35	22.06	22.0713274	250.381	250.509566	0.0113274	0.05134814	7.12833E-06
8	12.54	21.75	21.7584635	272.745	272.851132	0.00846349	0.03891258	3.97948E-06
9	13.73	21.45	21.4612626	294.5085	294.663135	0.01126257	0.05250616	7.04697E-06
10	15.73	21.09	20.9877416	331.7457	330.137175	0.10225845	0.48486699	0.000580933
11	17.02	20.68	20.6945094	351.9736	352.22055	0.01450943	0.07016165	1.16958E-05
12	19.11	20.22	20.230986	386.4042	386.614143	0.01098603	0.05433247	6.70515E-06
13	21.2	19.76	19.7709433	418.912	419.143998	0.01094331	0.05538114	6.65312E-06
14	23	19.36	19.3660248	445.28	445.41857	0.00602477	0.03111966	2.01655E-06
15	25.08	18.86	18.8664663	473.0088	473.170976	0.00646633	0.03428597	2.32297E-06
16	27.17	18.27	18.2747206	496.3959	496.524159	0.00472059	0.02583796	1.238E-06
17	28.06	17.95	17.9533108	503.677	503.769901	0.00331078	0.01844447	6.0896E-07
18	29.26	17.3	17.2928768	506.198	505.989576	0.00712316	0.04117432	2.81885E-06
**Average Value of different datasheets**						**0.01291347**	**0.06136339**	**3.61043E-05**

Significant are in value [bold].

In Table 3 result, Di-DE might adjust certain parameters, such as ξ₂ and ξ₃, to higher values to achieve a better overall fit. QUATRE, while effective, might converge to different regions of the parameter space due to its specific mutation and selection strategies, leading to different parameter values. In PEMFC models, parameters are often interdependent. A change in one parameter can be compensated by adjustments in others to achieve a similar or better fit to the experimental data. The higher values of ξ₂ and ξ₃ in the Di-DE algorithm may reflect such compensatory adjustments, optimizing the model’s predictive accuracy. Despite variations in individual parameter values, the Di-DE algorithm consistently achieves lower SSE values compared to other algorithms, including QUATRE. This indicates a superior fit to the experimental data, emphasizing the effectiveness of the Di-DE algorithm in capturing the underlying system dynamics. The Di-DE algorithm shows remarkable stability and consistency across multiple test scenarios, as evidenced by its minimal standard deviation in SSE values. This suggests that the Di-DE algorithm not only finds a good fit but does so reliably across different runs. The ultimate goal of parameter estimation is to accurately predict the V-I and P–V characteristics of the PEMFC. The Di-DE algorithm’s optimized parameters, even if numerically different, result in model predictions that closely match the experimental data, as shown in the performance metrics tables and characteristic curves (e.g., Fig. 2). The Di-DE algorithm’s ability to achieve lower SSE values and better model predictions, despite differences in individual parameters, demonstrates its robustness and effectiveness. The higher values of ξ₂ and ξ₃ in the Di-DE algorithm indicate its unique exploration of the parameter space, potentially uncovering more optimal regions that enhance the model’s predictive capability.

### PEMFC FC2

**Fig. 3:**
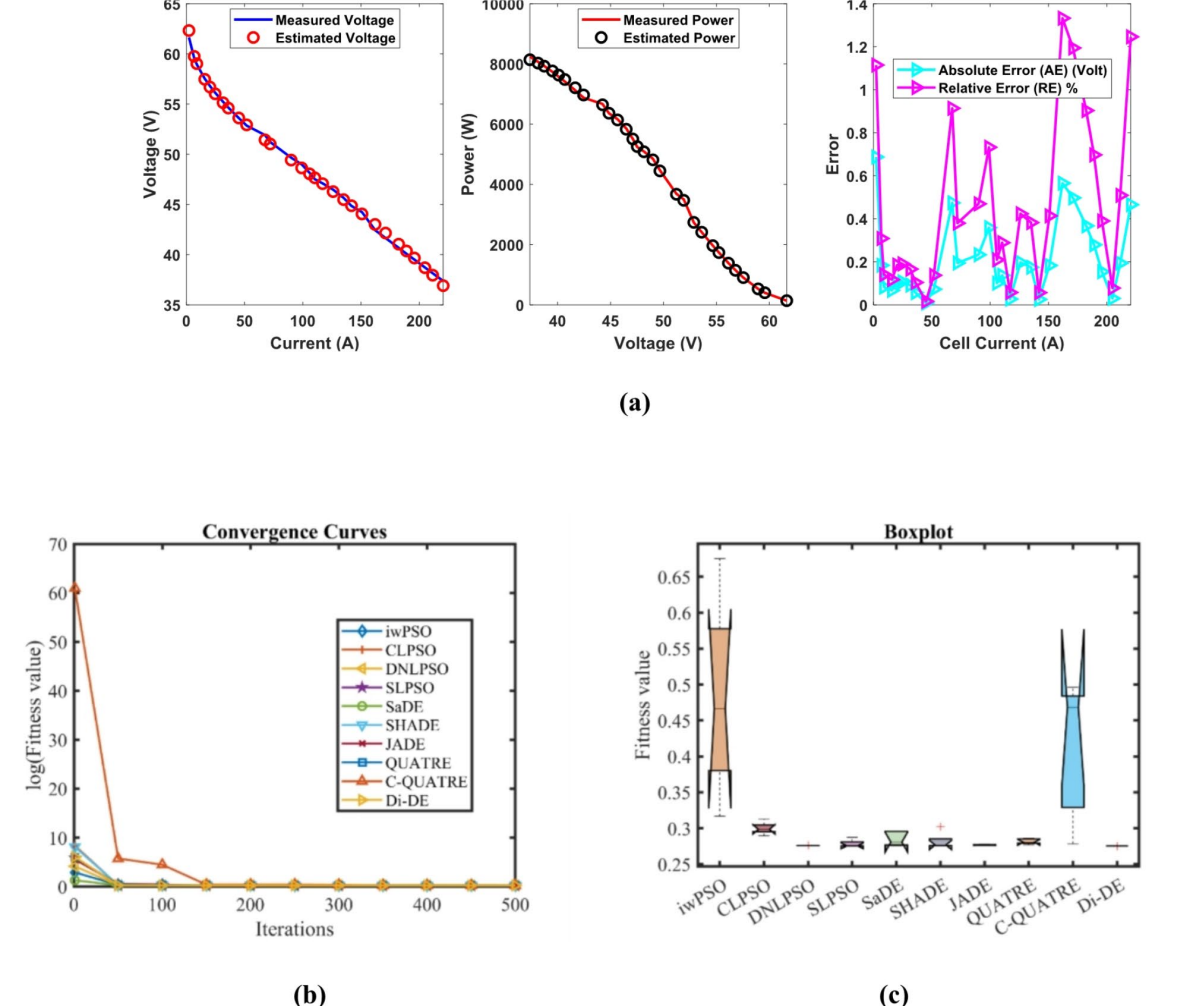
FD-DE Algorithm Characteristic curves of FC3; (**a**) V-I, P–V, and Error Curve, (**b**) Convergence Curve, (c) Box-Plo.

In Table 5, the Di-DE algorithm stands out for its exceptional stability, precision, and efficiency. The minimum, maximum, and mean values for Di-DE are remarkably consistent at 0.2752105, indicating no fluctuation and extraordinary consistency across multiple test scenarios. This minimal variation is further underscored by Di-DE standard deviation of just 3.10E-16, which is significantly lower than its closest competitor, JADE, with a standard deviation of 0.0006777. This stark contrast highlights Di-DE superior predictability and reliability in delivering stable results. In terms of runtime efficiency, Di-DE is unparalleled, recording a minimal 0.1367709 s, notably faster than other algorithms such as SaDE, which has a runtime of 8.9409815 s, demonstrating an efficiency improvement of approximately 98.47%. Compared to other algorithms, Di-DE consistently shows superior performance. For instance, iwPSO, with a minimum value of 0.3167561, has a much higher mean of 0.4810191 and a standard deviation of 0.1373454, indicating significant variability and less consistency. CLPSO and DNLPSO, while better than iwPSO, still exhibit higher mean values of 0.299904 and 0.2760286, respectively, and higher standard deviations of 0.0084425 and 7.19E-05, showing more variability. SLPSO, with a mean of 0.2784846 and standard deviation of 0.0052097, also falls short of Di-DE precision. SaDE, despite having a relatively low mean value of 0.2849208 and a standard deviation of 0.0101124, is much less efficient due to its significantly higher runtime. SHADE, with a mean of 0.28203 and a standard deviation of 0.0115292, shows more variability and less stability. JADE, while close to Di-DE with a mean of 0.2767057 and a very low standard deviation of 0.0006777, still exhibits slightly higher variability. QUATRE, despite its competitive mean of 0.2815882, has a higher standard deviation compared to Di-DE, indicating less consistency. C-QUATRE performs poorly with a high mean of 0.4137501 and a significant standard deviation of 0.0963235, indicating high variability and inconsistency. This detailed analysis reinforces in Tables 5, 6 and Fig. 3 shows Di-DE prowess in achieving optimal results with minimal computational overhead, superior stability, and efficiency compared to other algorithms, making it particularly valuable in applications where precision, reliability, and efficiency are paramount.

**Table 5:** Optimized parameters and optimal function value for FC2.

Algorithm	iwPSO	CLPSO	DNLPSO	SLPSO	SaDE	SHADE	JADE	QUATRE	C-QUATRE	Di-DE
${\xi }_{1}$	-1.1953721	-0.8532133	-1.19969	-1.1587662	-1.081411	-1.1457448	-1.0884226	-0.9553668	-1.19969	-0.8532
${\xi }_{2}$	0.0041216	0.0024136	0.004276	0.0033124	0.0033875	0.003278	0.0037124	0.0034544	0.0042813	0.0023983
${\xi }_{3}$	8.662E-05	3.677E-05	0.000098	3.767E-05	5.917E-05	3.781E-05	8.082E-05	8.999E-05	0.000098	3.6E-05
${\xi }_{4}$	-9.946E-05	-0.0000954	-0.0000954	-0.0000954	-9.542E-05	-0.0000954	-9.543E-05	-9.54E-05	-0.0000954	-0.0000954
$\lambda$	14.665117	15.660661	14	14	14.016483	14	14	14.162776	14	14
${R}_{c}$	0.0001766	0.0002242	0.0001	0.0001173	0.0001105	0.0001372	0.0001073	0.000141	0.0001448	0.0001204
*B*	0.0136	0.0180779	0.019593	0.0172423	0.0182395	0.0146644	0.0188174	0.016117	0.0141722	0.0167879
*Min*	0.3167561	0.2894785	0.2759	0.2752325	0.2756028	0.2757467	0.2759997	0.2769379	0.2781668	0.2752105
*Max*	0.6753932	0.3126621	0.2760607	0.2873687	0.296035	0.3024993	0.277795	0.2861245	0.4962589	0.2752105
*Mean*	0.4810191	0.299904	0.2760286	0.2784846	0.2849208	0.28203	0.2767057	0.2815882	0.4137501	0.2752105
*Std*	0.1373454	0.0084425	7.188E-05	0.0052097	0.0101124	0.0115292	0.0006777	0.0037171	0.0963235	3.103E-16
*RT*	4.6902869	4.7296291	4.1483967	4.4255948	8.9409815	4.7979299	4.7776205	5.3583937	9.2506389	0.1367709
*FR*	9.4	8	3.4	3.4	5.8	4.6	4	6.4	9	1

**Table 6:** Performance metrics of FD-DE Algorithm for FC2.

S. no.	*I*_*exp*_ (A)	*V*_*exp*_ (V)	*V*_*est*_ (V)	*P*_*exp*_ (W)	*P*_*est*_ (W)	*AE*_*v*_ (A)	*RE %*	*MBE*
1	2.25	61.64	62.327083	138.69	140.23594	0.6870834	1.1146713	0.0162787
2	6.75	59.57	59.753906	402.0975	403.33886	0.1839056	0.3087219	0.0011663
3	9	58.94	59.022995	530.46	531.20695	0.0829949	0.1408126	0.0002375
4	15.75	57.54	57.472447	906.255	905.19105	0.0675526	0.1174011	0.0001574
5	20.25	56.8	56.695006	1150.2	1148.0739	0.1049938	0.1848483	0.0003801
6	24.75	56.13	56.023038	1389.2175	1386.5702	0.1069625	0.1905621	0.0003945
7	31.5	55.23	55.138033	1739.745	1736.848	0.0919667	0.1665159	0.0002917
8	36	54.66	54.602993	1967.76	1965.7077	0.0570071	0.1042939	0.0001121
9	45	53.61	53.618863	2412.45	2412.8489	0.0088634	0.016533	2.709E-06
10	51.75	52.86	52.932643	2735.505	2739.2643	0.0726435	0.1374261	0.000182
11	67.5	51.91	51.435586	3503.925	3471.9021	0.4744138	0.9139161	0.007761
12	72	51.22	51.025394	3687.84	3673.8283	0.1946063	0.3799421	0.0013059
13	90	49.66	49.426717	4469.4	4448.4045	0.233283	0.4697604	0.0018766
14	99	49	48.641007	4851	4815.4597	0.3589933	0.7326394	0.004444
15	105.8	48.15	48.049163	5094.27	5083.6015	0.1008368	0.2094223	0.0003506
16	110.3	47.52	47.657396	5241.456	5256.6108	0.1373964	0.2891339	0.000651
17	117	47.1	47.07283	5510.7	5507.5211	0.0271704	0.0576866	2.546E-05
18	126	46.48	46.283057	5856.48	5831.6652	0.1969426	0.4237147	0.0013375
19	135	45.66	45.485304	6164.1	6140.516	0.1746963	0.3826025	0.0010524
20	141.8	44.85	44.875509	6359.73	6363.3472	0.025509	0.0568763	2.244E-05
21	150.8	44.24	44.056843	6671.392	6643.7719	0.183157	0.4140077	0.0011568
22	162	42.45	43.015692	6876.9	6968.542	0.5656916	1.3326069	0.0110347
23	171	41.66	42.15751	7123.86	7208.9342	0.4975097	1.1942143	0.008535
24	182.3	40.68	41.047506	7415.964	7482.9604	0.3675062	0.9034075	0.0046573
25	189	40.09	40.369538	7577.01	7629.8426	0.2795377	0.6972753	0.0026945
26	195.8	39.51	39.664127	7736.058	7766.2361	0.1541273	0.3900969	0.0008191
27	204.8	38.73	38.699832	7931.904	7925.7257	0.0301677	0.0778923	3.138E-05
28	211.5	38.15	37.955772	8068.725	8027.6458	0.1942281	0.5091168	0.0013008
29	220.5	37.38	36.914209	8242.29	8139.5832	0.4657905	1.2460956	0.0074814
**Average Value of different datasheets**						**0.2112254**	**0.4538688**	**0.0026118**

Significant are in value [bold].

### PEMFC FC3

**Fig. 4:**
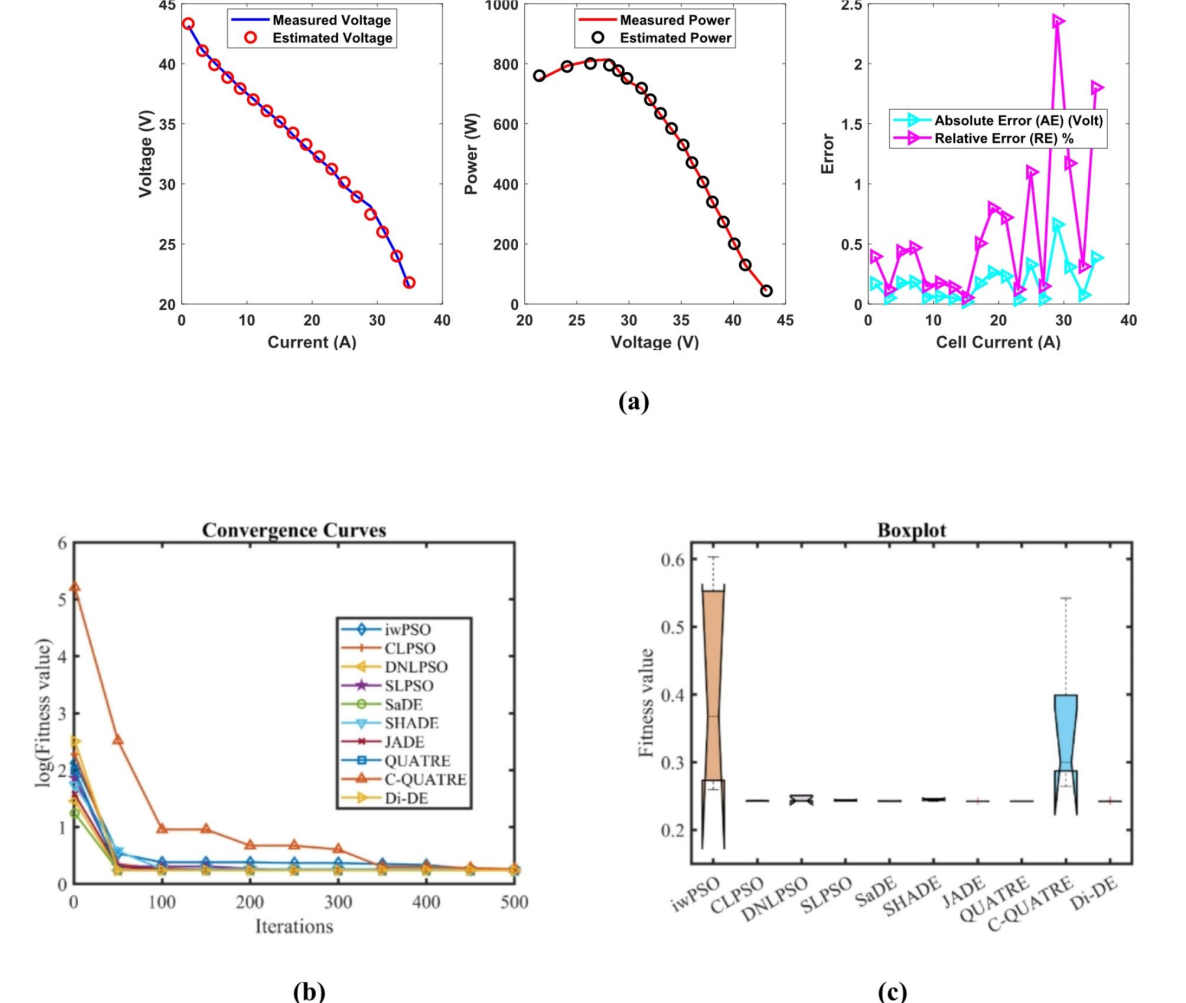
FD-DE Algorithm Characteristic curves of FC3; (**a**) V-I, P–V, and Error Curve, (**b**) Convergence Curve, (**c**) Box-Plot.

In Table 7, the Di-DE algorithm stands out for its exceptional stability, precision, and efficiency. The minimum, maximum, and mean values for Di-DE are remarkably consistent at 0.2422841, 0.2429272, and 0.2424127, respectively, indicating negligible fluctuation and extraordinary consistency across multiple test scenarios. This minimal variation is further underscored by Di-DE standard deviation of just 0.0002876, which is significantly lower than its closest competitor, JADE, with a standard deviation of 6.63E-05. This stark contrast highlights Di-DE superior predictability and reliability in delivering stable results. In terms of runtime efficiency, Di-DE is unparalleled, recording a minimal 0.0919761 s, notably faster than other algorithms such as SaDE, which has a runtime of 6.4015595 s, demonstrating an efficiency improvement of approximately 98.56%. Compared to other algorithms, Di-DE consistently shows superior performance. For instance, iwPSO, with a minimum value of 0.2597295, has a much higher mean of 0.4087695 and a standard deviation of 0.1540543, indicating significant variability and less consistency. CLPSO and DNLPSO, while better than iwPSO, still exhibit higher mean values of 0.2431374 and 0.2459765, respectively, and higher standard deviations of 0.0006814 and 0.0044766, showing more variability. SLPSO, with a mean of 0.2438719 and standard deviation of 0.0008112, also falls short of Di-DE precision. SaDE, despite having a relatively low mean value of 0.2425601 and a standard deviation of 0.0002438, is much less efficient due to its significantly higher runtime. SHADE, with a mean of 0.2448682 and a standard deviation of 0.0016061, shows more variability and less stability. JADE, while close to Di-DE with a mean of 0.2424112 and a very low standard deviation of 6.63E-05, still exhibits slightly higher variability. QUATRE, despite its competitive mean of 0.2424305, has a higher standard deviation compared to Di-DE, indicating less consistency. C-QUATRE performs poorly with a high mean of 0.3505867 and a significant standard deviation of 0.1116646, indicating high variability and inconsistency. This detailed analysis reinforces in Tables 7, 8 and Fig. 4 shows Di-DE prowess in achieving optimal results with minimal computational overhead, superior stability, and efficiency compared to other algorithms, making it particularly valuable in applications where precision, reliability, and efficiency are paramount.

**Table 7:** Optimized parameters and optimal function value for FC3.

Algorithm	iwPSO	CLPSO	DNLPSO	SLPSO	SaDE	SHADE	JADE	QUATRE	C-QUATRE	Di-DE
${\xi }_{1}$	-0.8576385	-1.1087346	-0.8532	-0.8923861	-1.025288	-0.955882	-1.0027376	-1.1342468	-0.925799	-0.8753941
${\xi }_{2}$	0.0031676	0.0033429	0.0032487	0.0026676	0.0036531	0.0026072	0.002962	0.0039686	0.0032646	0.0024728
${\xi }_{3}$	9.211E-05	5.297E-05	0.000098	5.267E-05	8.973E-05	0.000036	4.959E-05	8.831E-05	8.477E-05	4.347E-05
${\xi }_{4}$	-0.0000954	-0.0000954	-0.0000954	-0.0000954	-9.54E-05	-0.0000954	-0.0000954	-9.545E-05	-9.92E-05	-0.0000954
$\lambda$	22.742041	17.602246	23	14.309877	22.887143	20.610971	21.809067	22.052643	14.753272	23
${R}_{c}$	0.0001	0.0006535	0.0006726	0.0005627	0.0006522	0.0006902	0.0006665	0.0006444	0.0002795	0.0006726
*B*	0.1892879	0.1737116	0.1753203	0.1732492	0.1756973	0.1744361	0.1752027	0.1755681	0.1736609	0.1753203
*Min*	0.2597295	0.2426293	0.2422841	0.242982	0.2422986	0.2424781	0.2423458	0.2423403	0.2641276	0.2422841
*Max*	0.6031484	0.2443066	0.2508719	0.2449078	0.2429055	0.2464987	0.2425229	0.2425011	0.54231	0.2429272
*Mean*	0.4087695	0.2431374	0.2459765	0.2438719	0.2425601	0.2448682	0.2424112	0.2424305	0.3505867	0.2424127
*Std*	0.1540543	0.0006814	0.0044766	0.0008112	0.0002438	0.0016061	6.628E-05	6.252E-05	0.1116646	0.0002876
*RT*	3.4818246	3.3511308	2.8428073	3.0580569	6.4015595	3.4298999	4.7525351	6.7554784	12.462061	0.0919761
*FR*	9.8	5.4	5.6	6.8	3.6	6.8	2.8	3	9.2	2

**Table 8:** Performance metrics of FD-DE Algorithm for FC3.

S. no.	*I*_*exp*_ (A)	*V*_*exp*_ (V)	*V*_*est*_ (V)	*P*_*exp*_ (W)	*P*_*est*_ (W)	*AE*_*v*_ (A)	*RE %*	*MBE*
1	1.004	43.17	43.340809	43.34268	43.514172	0.1708093	0.3956666	0.0016209
2	3.166	41.14	41.090078	130.24924	130.09119	0.0499223	0.1213474	0.0001385
3	5.019	40.09	39.914512	201.21171	200.33094	0.1754879	0.4377349	0.0017109
4	7.027	39.04	38.857152	274.33408	273.04921	0.1828479	0.4683603	0.0018574
5	8.958	37.99	37.933465	340.31442	339.80798	0.0565353	0.1488162	0.0001776
6	10.97	37.08	37.014537	406.7676	406.04947	0.0654633	0.176546	0.0002381
7	13.05	36.03	36.079906	470.1915	470.84277	0.0499056	0.1385111	0.0001384
8	15.06	35.19	35.171364	529.9614	529.68074	0.018636	0.0529583	1.929E-05
9	17.07	34.07	34.242088	581.5749	584.51245	0.1720883	0.5051022	0.0016452
10	19.07	33.02	33.283126	629.6914	634.70921	0.263126	0.7968686	0.0038464
11	21.08	32.04	32.2707	675.4032	680.26636	0.2307002	0.7200381	0.0029568
12	23.01	31.2	31.237694	717.912	718.77933	0.0376937	0.120813	7.893E-05
13	24.94	29.8	30.127372	743.212	751.37665	0.3273715	1.0985621	0.005954
14	26.87	28.96	28.917134	778.1552	777.00339	0.0428661	0.1480181	0.0001021
15	28.96	28.12	27.457757	814.3552	795.17664	0.6622432	2.3550612	0.0243648
16	30.81	26.3	25.991805	810.303	800.8075	0.3081955	1.171846	0.0052769
17	32.97	24.06	23.984869	793.2582	790.78113	0.0751311	0.3122654	0.0003136
18	34.9	21.4	21.785634	746.86	760.31862	0.3856339	1.8020275	0.0082619
**Average Value of different datasheets**						**0.1819254**	**0.6094746**	**0.0032612**

Significant are in value [bold].

### PEMFC FC4

**Fig. 5:**
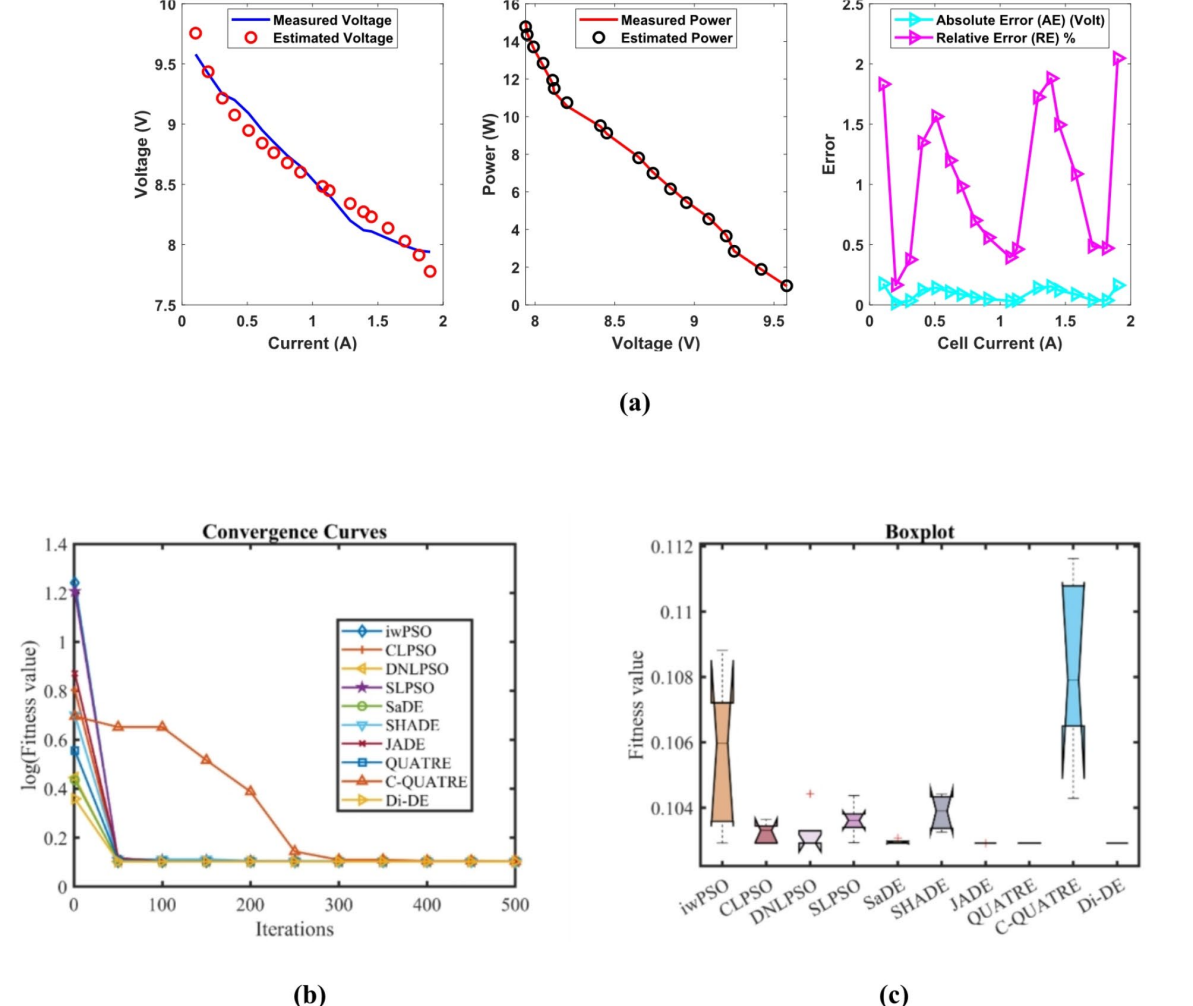
FD-DE Algorithm Characteristic curves of FC4; (**a**) V-I, P–V, and Error Curve, (**b**) Convergence Curve, (**c**) Box-Plot.

In Table 9, the Di-DE algorithm stands out for its exceptional stability, precision, and efficiency. The minimum, maximum, and mean values for Di-DE are remarkably consistent at 0.1029149, indicating no fluctuation and extraordinary consistency across multiple test scenarios. This minimal variation is further underscored by Di-DE standard deviation of just 6.90E-17, which is significantly lower than its closest competitor, JADE, with a standard deviation of 9.19E-07. This stark contrast highlights Di-DE superior predictability and reliability in delivering stable results. In terms of runtime efficiency, Di-DE is unparalleled, recording a minimal 0.1038601 s, notably faster than other algorithms such as SaDE, which has a runtime of 9.7863818 s, demonstrating an efficiency improvement of approximately 98.94%. Compared to other algorithms, Di-DE consistently shows superior performance. For instance, iwPSO, with a minimum value of 0.1029195, has a much higher mean of 0.105637 and a standard deviation of 0.0023473, indicating significant variability and less consistency. CLPSO and DNLPSO, while better than iwPSO, still exhibit higher mean values of 0.1032299 and 0.1032174, respectively, and higher standard deviations of 0.0003137 and 0.0006766, showing more variability. SLPSO, with a mean of 0.103616 and standard deviation of 0.0005119, also falls short of Di-DE precision. SaDE, despite having a relatively low mean value of 0.1029575 and a standard deviation of 6.57E-05, is much less efficient due to its significantly higher runtime. SHADE, with a mean of 0.1038555 and a standard deviation of 0.000521, shows more variability and less stability. JADE, while close to Di-DE with a mean of 0.1029156 and a very low standard deviation of 9.19E-07, still exhibits slightly higher variability. QUATRE, despite its competitive mean of 0.1029171, has a higher standard deviation compared to Di-DE, indicating less consistency. C-QUATRE performs poorly with a high mean of 0.1083083 and a significant standard deviation of 0.0028879, indicating high variability and inconsistency. This detailed analysis reinforces in Tables 9, 10 and Fig. 5 shows Di-DE prowess in achieving optimal results with minimal computational overhead, superior stability, and efficiency compared to other algorithms, making it particularly valuable in applications where precision, reliability, and efficiency are paramount.

**Table 9:** Optimized parameters and optimal function value for FC4.

Algorithm	iwPSO	CLPSO	DNLPSO	SLPSO	SaDE	SHADE	JADE	QUATRE	C-QUATRE	Di-DE
${\xi }_{1}$	-1.0873361	-0.8532	-0.8532	-1.186609	-0.9121892	-1.189796	-0.8642168	-0.8861256	-1.134611	-0.8533017
${\xi }_{2}$	0.0025395	0.0017933	0.002372	0.0026361	0.0020899	0.0027812	0.0022595	0.0017783	0.0025925	0.0016686
${\xi }_{3}$	5.796E-05	5.645E-05	0.000098	4.284E-05	6.463E-05	5.26E-05	8.747E-05	4.805E-05	5.144E-05	4.747E-05
${\xi }_{4}$	-0.0001113	-0.0001113	-0.0001113	-0.0001112	-0.0001114	-0.000112	-0.0001113	-0.0001114	-0.0001117	-0.0001113
$\lambda$	14	14	14	14.021574	14.000371	15.047507	14	14.000389	19.760395	14
${R}_{c}$	0.0008	0.0008	0.0008	0.0007942	0.0008	0.0007513	0.0008	0.0007995	0.0006856	0.0008
*B*	0.0136	0.0136	0.0136	0.0136	0.0136008	0.0136186	0.0136	0.0136005	0.01425	0.0136
*Min*	0.1029195	0.1029149	0.1029149	0.1029292	0.1029152	0.1032493	0.102915	0.1029157	0.1042816	0.1029149
*Max*	0.1088139	0.1036404	0.1044277	0.1043723	0.1030727	0.104413	0.1029172	0.1029192	0.1116228	0.1029149
*Mean*	0.105637	0.1032299	0.1032174	0.103616	0.1029575	0.1038555	0.1029156	0.1029171	0.1083083	0.1029149
*Std*	0.0023473	0.0003137	0.0006766	0.0005119	6.572E-05	0.000521	9.194E-07	1.437E-06	0.0028879	6.904E-17
*RT*	4.9774322	4.6279035	3.0937258	3.3021467	9.7863818	4.0884796	5.4645914	6.3019192	12.535487	0.1038601
*FR*	8.6	5	2.8	7.2	5.2	7.8	3.6	4.2	9.2	1.4

**Table 10:** Performance metrics of FD-DE Algorithm for FC4.

S. no.	***I***_***exp***_** (A)**	***V***_***exp***_** (V)**	***V***_***est***_** (V)**	***P***_***exp***_** (W)**	***P***_***est***_** (W)**	***AE***_***v***_** (A)**	***RE %***	***MBE***
1	0.104	9.58	9.7555317	0.99632	1.0145753	0.1755317	1.8322722	0.0017117
2	0.2	9.42	9.4355344	1.884	1.8871069	0.0155344	0.1649086	1.341E-05
3	0.309	9.25	9.215306	2.85825	2.8475296	0.034694	0.3750699	6.687E-05
4	0.403	9.2	9.0759951	3.7076	3.657626	0.1240049	1.3478794	0.0008543
5	0.51	9.09	8.9478926	4.6359	4.5634252	0.1421074	1.5633379	0.0011219
6	0.614	8.95	8.8427145	5.4953	5.4294267	0.1072855	1.1987202	0.0006395
7	0.703	8.85	8.7628613	6.22155	6.1602915	0.0871387	0.9846183	0.0004218
8	0.806	8.74	8.6786854	7.04444	6.9950205	0.0613146	0.7015398	0.0002089
9	0.908	8.65	8.6015874	7.8542	7.8102414	0.0484126	0.5596829	0.0001302
10	1.076	8.45	8.4833936	9.0922	9.1281315	0.0333936	0.3951901	6.195E-05
11	1.127	8.41	8.4488673	9.47807	9.5218734	0.0388673	0.4621558	8.393E-05
12	1.288	8.2	8.3413839	10.5616	10.743702	0.1413839	1.7241938	0.0011105
13	1.39	8.12	8.2726626	11.2868	11.499001	0.1526626	1.8800809	0.0012948
14	1.45	8.11	8.2311985	11.7595	11.935238	0.1211985	1.4944324	0.0008161
15	1.578	8.05	8.1375146	12.7029	12.840998	0.0875146	1.0871382	0.0004255
16	1.707	7.99	8.0288558	13.63893	13.705257	0.0388558	0.4863057	8.388E-05
17	1.815	7.95	7.9126024	14.42925	14.361373	0.0373976	0.4704101	7.77E-05
18	1.9	7.94	7.7774129	15.086	14.777085	0.1625871	2.0476959	0.0014686
**Average Value of different datasheets**						**0.089438**	**1.0430907**	**0.0005884**

Significant are in value [bold].

### PEMFC FC5

**Fig. 6:**
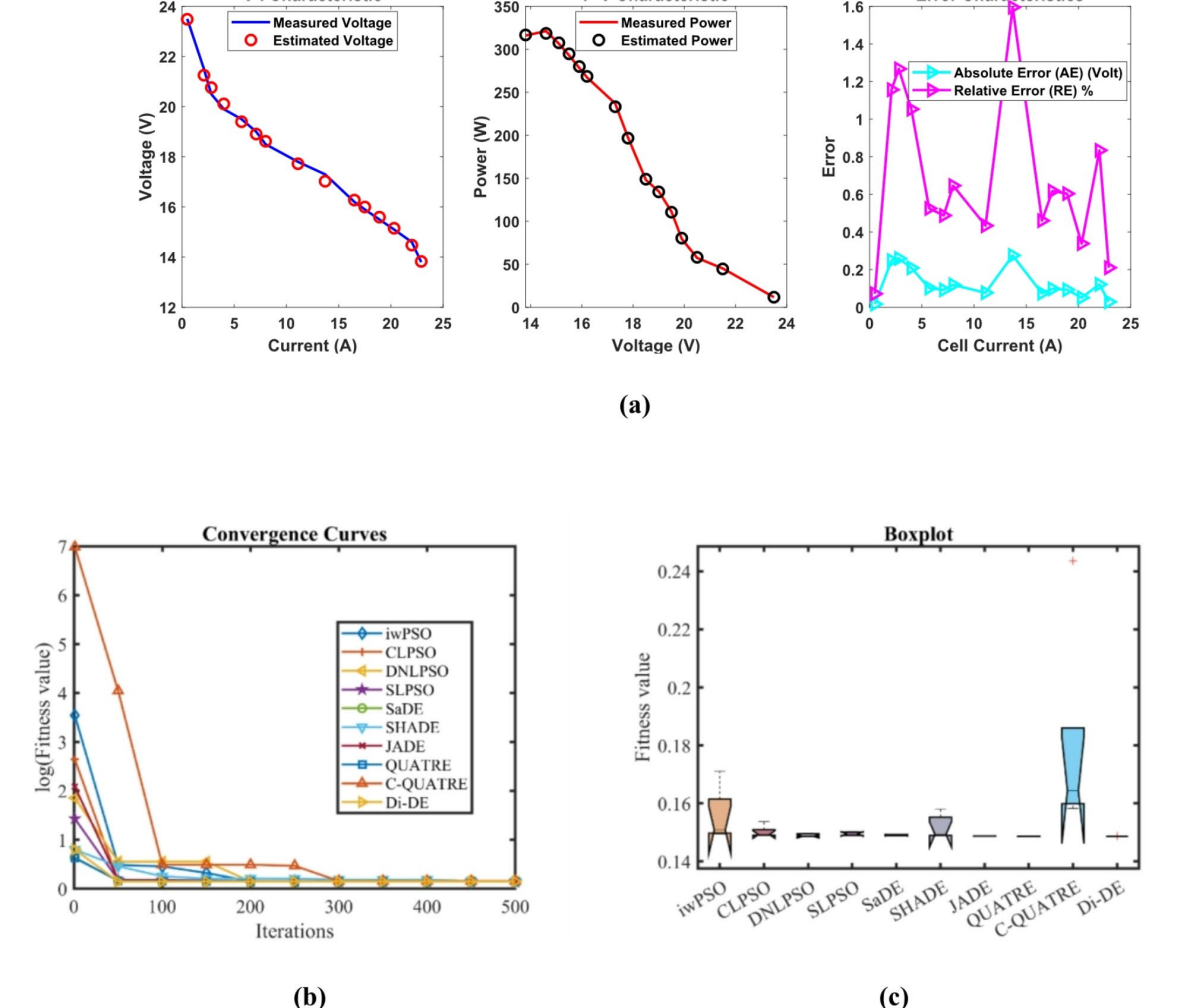
FD-DE Algorithm Characteristic curves of FC5; (**a**) V-I, P–V, and Error Curve, (**b**) Convergence Curve, (**c**) Box-Plot.

In Table 11, the Di-DE algorithm stands out for its exceptional stability, precision, and efficiency. The minimum, maximum, and mean values for Di-DE are remarkably consistent at 0.1486318, indicating no fluctuation and extraordinary consistency across multiple test scenarios. This minimal variation is further underscored by Di-DE standard deviation of just 6.62E-16, which is significantly lower than its closest competitor, JADE, with a standard deviation of 5.81E-05. This stark contrast highlights Di-DE superior predictability and reliability in delivering stable results. In terms of runtime efficiency, Di-DE is unparalleled, recording a minimal 0.0883434 s, notably faster than other algorithms such as SaDE, which has a runtime of 8.308184 s, demonstrating an efficiency improvement of approximately 98.94%. Compared to other algorithms, Di-DE consistently shows superior performance. For instance, iwPSO, with a minimum value of 0.1494508, has a much higher mean of 0.1558602 and a standard deviation of 0.0091958, indicating significant variability and less consistency. CLPSO and DNLPSO, while better than iwPSO, still exhibit higher mean values of 0.1501683 and 0.1490179, respectively, and higher standard deviations of 0.0020006 and 0.0005245, showing more variability. SLPSO, with a mean of 0.1494863 and standard deviation of 0.0006261, also falls short of Di-DE precision. SaDE, despite having a relatively low mean value of 0.1489959 and a standard deviation of 0.0002324, is much less efficient due to its significantly higher runtime. SHADE, with a mean of 0.1518655 and a standard deviation of 0.0041443, shows more variability and less stability. JADE, while close to Di-DE with a mean of 0.1487312 and a very low standard deviation of 5.81E-05, still exhibits slightly higher variability. QUATRE, despite its competitive mean of 0.1486549, has a higher standard deviation compared to Di-DE, indicating less consistency. C-QUATRE performs poorly with a high mean of 0.178701 and a significant standard deviation of 0.0364576, indicating high variability and inconsistency. This detailed analysis reinforces in Tables 11, 12 and Fig. 6 shows Di-DE prowess in achieving optimal results with minimal computational overhead, superior stability, and efficiency compared to other algorithms, making it particularly valuable in applications where precision, reliability, and efficiency are paramount.

**Table 11:** Optimized parameters and optimal function value for FC5.

Algorithm	iwPSO	CLPSO	DNLPSO	SLPSO	SaDE	SHADE	JADE	QUATRE	C-QUATRE	Di-DE
${\xi }_{1}$	-1.19969	-1.1236524	-0.8532	-0.8860452	-1.100897	-0.8949807	-1.1601875	-0.9485128	-0.9793038	-1.0843287
${\xi }_{2}$	0.0036437	0.0031127	0.0030207	0.0029067	0.0035212	0.0024001	0.003733	0.0027545	0.0034953	0.003475
${\xi }_{3}$	6.271E-05	4.065E-05	9.037E-05	7.541E-05	7.458E-05	3.736E-05	7.729E-05	5.154E-05	0.000098	7.47E-05
${\xi }_{4}$	-0.0001712	-0.0001739	-0.0001737	-0.0001738	-0.0001736	-0.0001741	-0.0001742	-0.0001742	-0.0001792	-0.0001739
$\lambda$	14.345071	14.980515	14.355314	14.758105	14.539459	14.440657	14.439053	14.509577	17.112427	14.439129
${R}_{c}$	0.0002492	0.0002709	0.0001	0.0001682	0.0001168	0.0001293	0.0001037	0.0001001	0.0006624	0.0001
*B*	0.0136	0.0141966	0.0136	0.0142672	0.0140036	0.0136	0.0137614	0.0139298	0.0136	0.013795
*Min*	0.1494508	0.148914	0.1486427	0.1487993	0.1486901	0.1487797	0.1486753	0.1486384	0.1582661	0.1486318
*Max*	0.170989	0.1536727	0.1497299	0.1502376	0.1492305	0.1580491	0.1488291	0.1486851	0.2436448	0.1486318
*Mean*	0.1558602	0.1501683	0.1490179	0.1494863	0.1489959	0.1518655	0.1487312	0.1486549	0.178701	0.1486318
*Std*	0.0091958	0.0020006	0.0005245	0.0006261	0.0002324	0.0041443	5.81E-05	2.181E-05	0.0364576	6.622E-16
*RT*	3.2925955	3.4457436	5.016824	3.0759218	8.308184	3.9657438	3.4032798	4.1547653	11.134975	0.0883434
*FR*	8.6	6.8	4.4	6.2	5.4	6.8	3.8	2.2	9.8	1

**Table 12:** Performance metrics of FD-DE Algorithm for FC5.

S. no.	***I***_***exp***_** (A)**	***V***_***exp***_** (V)**	***V***_***est***_** (V)**	***P***_***exp***_** (W)**	***P***_***est***_** (W)**	***AE***_***v***_** (A)**	***RE %***	***MBE***
1	0.5	23.5	23.483086	11.75	11.741543	0.016914	0.0719746	1.907E-05
2	2.1	21.5	21.251304	45.15	44.627738	0.2486963	1.1567269	0.0041233
3	2.8	20.5	20.759815	57.4	58.127481	0.2598148	1.2673893	0.0045002
4	4	19.9	20.109577	79.6	80.438309	0.2095771	1.0531515	0.0029282
5	5.7	19.5	19.397532	111.15	110.56593	0.102468	0.5254769	0.0007
6	7.1	19	18.907254	134.9	134.2415	0.0927465	0.4881392	0.0005735
7	8	18.5	18.61964	148	148.95712	0.1196404	0.646705	0.0009543
8	11.1	17.8	17.722754	197.58	196.72256	0.0772464	0.4339687	0.0003978
9	13.7	17.3	17.024089	237.01	233.23001	0.2759114	1.5948634	0.0050751
10	16.5	16.2	16.274644	267.3	268.53162	0.0746437	0.4607633	0.0003714
11	17.5	15.9	15.99828	278.25	279.96991	0.0982804	0.6181156	0.0006439
12	18.9	15.5	15.593658	292.95	294.72014	0.093658	0.6042451	0.0005848
13	20.3	15.1	15.15114	306.53	307.56814	0.0511398	0.338674	0.0001744
14	22	14.6	14.478187	321.2	318.52011	0.121813	0.8343358	0.0009892
15	22.9	13.8	13.829041	316.02	316.68505	0.0290413	0.2104444	5.623E-05
**Average Value of different datasheets**						**0.1247727**	**0.6869982**	**0.0014728**

Significant are in value [bold].

### PEMFC FC6

**Fig. 7:**
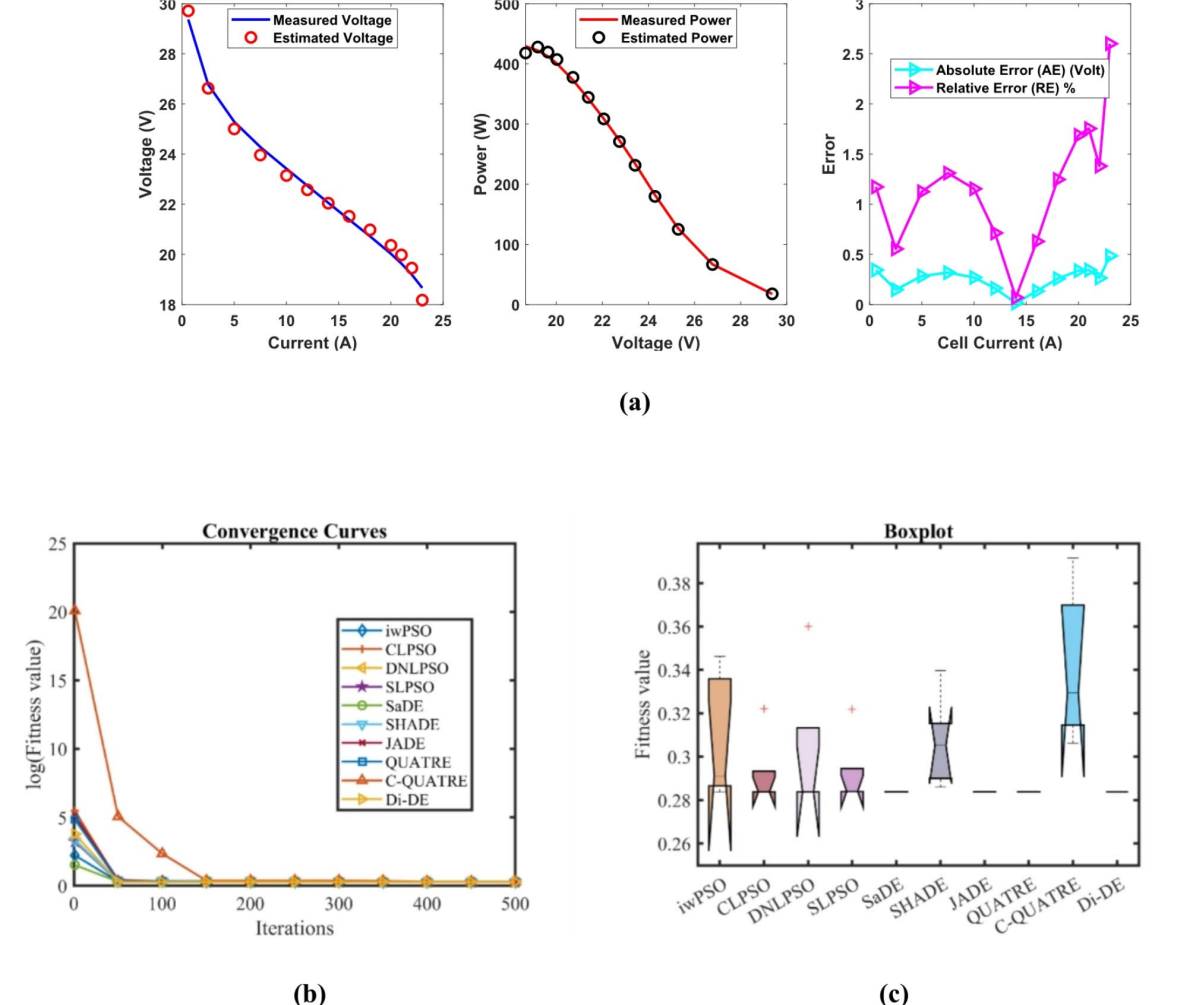
FD-DE Algorithm Characteristic curves of FC6; (**a**) V-I, P–V, and Error Curve, (**b**) Convergence Curve, (**c**) Box-Plot.

In Table 13, the Di-DE algorithm stands out for its exceptional stability, precision, and efficiency. The minimum, maximum, and mean values for Di-DE are remarkably consistent at 0.2837738, indicating no fluctuation and extraordinary consistency across multiple test scenarios. This minimal variation is further underscored by Di-DE standard deviation of just 1.55E-16, which is significantly lower than its closest competitor, JADE, with a standard deviation of 4.05E-05. This stark contrast highlights Di-DE superior predictability and reliability in delivering stable results. In terms of runtime efficiency, Di-DE is unparalleled, recording a minimal 0.0775022 s, notably faster than other algorithms such as SaDE, which has a runtime of 6.744323 s, demonstrating an efficiency improvement of approximately 98.85%. Compared to other algorithms, Di-DE consistently shows superior performance. For instance, iwPSO, with a minimum value of 0.2838017, has a much higher mean of 0.3082655 and a standard deviation of 0.0289726, indicating significant variability and less consistency. CLPSO and DNLPSO, while better than iwPSO, still exhibit higher mean values of 0.2914463 and 0.3018187, respectively, and higher standard deviations of 0.0171524 and 0.0331225, showing more variability. SLPSO, with a mean of 0.2918697 and standard deviation of 0.0168379, also falls short of Di-DE precision. SaDE, despite having a relatively low mean value of 0.2838064 and a standard deviation of 4.10E-05, is much less efficient due to its significantly higher runtime. SHADE, with a mean of 0.3059039 and a standard deviation of 0.0209494, shows more variability and less stability. JADE, while close to Di-DE with a mean of 0.2838434 and a very low standard deviation of 4.05E-05, still exhibits slightly higher variability. QUATRE, despite its competitive mean of 0.2837953, has a higher standard deviation compared to Di-DE, indicating less consistency. C-QUATRE performs poorly with a high mean of 0.3414938 and a significant standard deviation of 0.0351161, indicating high variability and inconsistency. This detailed analysis reinforces in Tables 13, 14 and Fig. 7 shows Di-DE prowess in achieving optimal results with minimal computational overhead, superior stability, and efficiency compared to other algorithms, making it particularly valuable in applications where precision, reliability, and efficiency are paramount.

**Table 13:** Optimized parameters and optimal function value for FC6.

Algorithm	iwPSO	CLPSO	DNLPSO	SLPSO	SaDE	SHADE	JADE	QUATRE	C-QUATRE	Di-DE
${\xi }_{1}$	-1.0238537	-0.9648569	-1.0220512	-0.8862365	-0.9708129	-1.1794329	-0.8790866	-0.9517368	-1.0927263	-0.8556916
${\xi }_{2}$	0.0026172	0.0023523	0.003244	0.0025174	0.0030607	0.0032355	0.0023328	0.0026758	0.0027021	0.0020644
${\xi }_{3}$	5.279E-05	4.638E-05	0.000098	7.474E-05	9.574E-05	6.426E-05	6.314E-05	7.226E-05	4.387E-05	4.888E-05
${\xi }_{4}$	-0.0001698	-0.0001697	-0.0001697	-0.0001704	-0.0001697	-0.0001672	-0.0001696	-0.0001697	-0.0001791	-0.0001697
$\lambda$	14	14	14	14	14	14.057142	14	14.00102	16.150394	14
${R}_{c}$	0.0008	0.0008	0.0008	0.0008	0.0008	0.0007963	0.0008	0.0008	0.0006786	0.0008
*B*	0.0173742	0.0173206	0.0173175	0.0172028	0.0173171	0.0182336	0.0173736	0.0173453	0.0160712	0.0173175
*Min*	0.2838017	0.2837738	0.2837738	0.2838699	0.2837738	0.2859862	0.2837998	0.2837793	0.3062681	0.2837738
*Max*	0.3463309	0.3221295	0.360081	0.3219684	0.2838649	0.3397528	0.2839076	0.2838156	0.3916348	0.2837738
*Mean*	0.3082655	0.2914463	0.3018187	0.2918697	0.2838064	0.3059039	0.2838434	0.2837953	0.3414938	0.2837738
*Std*	0.0289726	0.0171524	0.0331225	0.0168379	4.098E-05	0.0209494	4.048E-05	1.348E-05	0.0351161	1.545E-16
*RT*	4.8957224	5.4537933	2.4035455	4.5313337	6.744323	2.8621312	2.9966077	6.1499943	10.926255	0.0775022
*FR*	8	4	4.4	6.8	4	8.2	5.4	3.8	9.4	1

**Table 14:** Performance metrics of FD-DE Algorithm for FC6.

S. no.	***I***_***exp***_** (A)**	***V***_***exp***_** (V)**	***V***_***est***_** (V)**	***P***_***exp***_** (W)**	***P***_***est***_** (W)**	***AE***_***v***_** (A)**	***RE %***	***MBE***
1	0.6	29.37	29.714698	17.622	17.828819	0.344698	1.1736396	0.0091397
2	2.5	26.77739	26.628794	66.943475	66.571985	0.1485961	0.5549311	0.0016985
3	5	25.29025	25.005587	126.45125	125.02793	0.2846631	1.1255845	0.0062333
4	7.5	24.281859	23.96352	182.11394	179.7264	0.3183385	1.3110139	0.0077953
5	10	23.418	23.147545	234.18	231.47545	0.2704551	1.1549028	0.0056266
6	12	22.739103	22.576729	272.86924	270.92075	0.1623736	0.7140721	0.0020281
7	14	22.058523	22.043056	308.81932	308.60279	0.0154667	0.0701168	1.84E-05
8	16	21.386148	21.520882	342.17837	344.33412	0.1347343	0.6300072	0.0013964
9	18	20.721728	20.980157	372.9911	377.64282	0.2584287	1.247139	0.0051373
10	20	20.026	20.363999	400.52	407.27999	0.3379994	1.6878029	0.008788
11	21	19.63635	19.980915	412.36335	419.59921	0.344565	1.7547302	0.0091327
12	22	19.191807	19.456783	422.21975	428.04923	0.264976	1.3806726	0.0054009
13	23	18.66363	18.178122	429.26349	418.0968	0.4855082	2.6013597	0.0181322
**Average Value of different datasheets**						**0.2592925**	**1.1850748**	**0.0061944**

Significant are in value [bold].

### PEMFC FC7

**Fig. 8:**
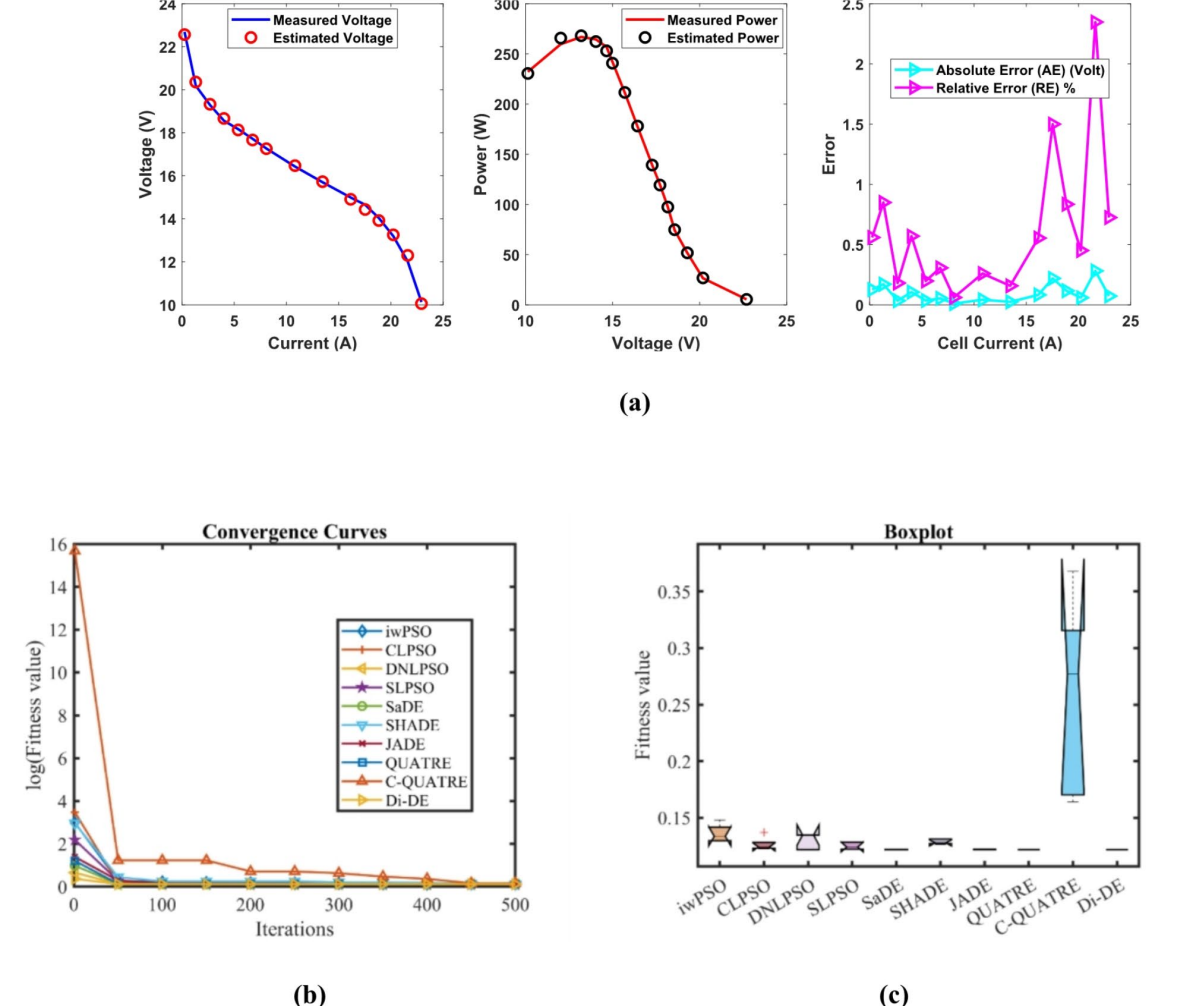
FD-DE Algorithm Characteristic curves of FC7; (**a**) V-I, P–V, and Error Curve, (**b**) Convergence Curve, (**c**) Box-Plot.

In Table 15, the Di-DE algorithm stands out for its exceptional stability, precision, and efficiency. The minimum, maximum, and mean values for Di-DE are remarkably consistent at 0.1217552, indicating no fluctuation and extraordinary consistency across multiple test scenarios. This minimal variation is further underscored by Di-DE standard deviation of just 1.75E-16, which is significantly lower than its closest competitor, JADE, with a standard deviation of 0.0002177. This stark contrast highlights Di-DE superior predictability and reliability in delivering stable results. In terms of runtime efficiency, Di-DE is unparalleled, recording a minimal 0.0841613 s, notably faster than other algorithms such as SaDE, which has a runtime of 7.8484291 s, demonstrating an efficiency improvement of approximately 98.93%. Compared to other algorithms, Di-DE consistently shows superior performance. For instance, iwPSO, with a minimum value of 0.1292194, has a much higher mean of 0.1360529 and a standard deviation of 0.0078242, indicating significant variability and less consistency. CLPSO and DNLPSO, while better than iwPSO, still exhibit higher mean values of 0.1264454 and 0.1295327, respectively, and higher standard deviations of 0.0059507 and 0.0070999, showing more variability. SLPSO, with a mean of 0.1251227 and standard deviation of 0.0032349, also falls short of Di-DE precision. SaDE, despite having a relatively low mean value of 0.1218707 and a standard deviation of 0.0001289, is much less efficient due to its significantly higher runtime. SHADE, with a mean of 0.1288482 and a standard deviation of 0.002251, shows more variability and less stability. JADE, while close to Di-DE with a mean of 0.1220313 and a very low standard deviation of 0.0002177, still exhibits slightly higher variability. QUATRE, despite its competitive mean of 0.1217815, has a higher standard deviation compared to Di-DE, indicating less consistency. C-QUATRE performs poorly with a high mean of 0.255831 and a significant standard deviation of 0.0868443, indicating high variability and inconsistency. This detailed analysis reinforces in Tables 15, 16 and Fig. 8 shows Di-DE prowess in achieving optimal results with minimal computational overhead, superior stability, and efficiency compared to other algorithms, making it particularly valuable in applications where precision, reliability, and efficiency are paramount.

**Table 15:** Optimized parameters and optimal function value for FC7.

Algorithm	iwPSO	CLPSO	DNLPSO	SLPSO	SaDE	SHADE	JADE	QUATRE	C-QUATRE	Di-DE
${\xi }_{1}$	-1.1230446	-0.9622942	-1.19969	-1.158016	-1.0398275	-1.1215313	-0.9335995	-1.1902448	-0.9987489	-1.1996898
${\xi }_{2}$	0.0033145	0.0024759	0.0038657	0.0029238	0.0031197	0.0030595	0.0025656	0.0032522	0.0028173	0.003169
${\xi }_{3}$	7.429E-05	4.673E-05	0.000098	3.772E-05	7.743E-05	5.529E-05	5.943E-05	5.494E-05	6.458E-05	4.681E-05
${\xi }_{4}$	-0.000143	-0.0001487	-0.0001493	-0.0001487	-0.0001493	-0.0001483	-0.0001493	-0.0001493	-0.0001391	-0.0001493
$\lambda$	20.80354	22.626937	23	22.994167	22.999825	22.775269	23	22.99908	17.807943	23
${R}_{c}$	0.0001128	0.0001129	0.0001	0.000104	0.0001	0.0003493	0.0001	0.0001001	0.0003507	0.0001
*B*	0.0501384	0.0507094	0.0509795	0.0512041	0.0509623	0.0495228	0.0510527	0.0509416	0.0456102	0.0509795
*Min*	0.1292194	0.1224303	0.1217552	0.1219449	0.1217569	0.1264157	0.121798	0.1217606	0.1639571	0.1217552
*Max*	0.1479443	0.1369281	0.1347178	0.1287928	0.1220724	0.1313541	0.122288	0.1218038	0.3677498	0.1217552
*Mean*	0.1360529	0.1264454	0.1295327	0.1251227	0.1218707	0.1288482	0.1220313	0.1217815	0.255831	0.1217552
*Std*	0.0078242	0.0059507	0.0070999	0.0032349	0.0001289	0.002251	0.0002177	1.659E-05	0.0868443	1.747E-16
*RT*	3.7709951	2.9000432	2.5704719	4.6468682	7.8484291	3.4367339	3.6864411	4.4248832	9.8974862	0.0841613
*FR*	8.4	6.4	5.8	6	3.4	7.2	4	2.8	10	1

**Table 16:** Performance metrics of FD-DE algorithm for FC7.

**S. no.**	***I***_***exp***_** (A)**	***V***_***exp***_** (V)**	***V***_***est***_** (V)**	***P***_***exp***_** (W)**	***P***_***est***_** (W)**	***AE***_***v***_** (A)**	***RE %***	***MBE***
1	0.2417	22.6916	22.564587	5.4845597	5.4538606	0.1270131	0.5597364	0.0010755
2	1.3177	20.1869	20.358455	26.600278	26.826336	0.1715549	0.8498328	0.0019621
3	2.6819	19.2897	19.324645	51.733046	51.826766	0.0349452	0.1811601	8.141E-05
4	4.0118	18.5607	18.666642	74.461816	74.886835	0.1059421	0.5707871	0.0007482
5	5.3755	18.1682	18.13216	97.663159	97.469426	0.0360401	0.198369	8.659E-05
6	6.7563	17.7196	17.665131	119.71893	119.35092	0.0544694	0.3073964	0.0001978
7	8.0689	17.271	17.260392	139.35797	139.27238	0.0106078	0.06142	7.502E-06
8	10.8134	16.4299	16.472653	177.66308	178.12538	0.0427526	0.260212	0.0001219
9	13.4556	15.7009	15.72573	211.26503	211.59914	0.0248304	0.1581462	4.11E-05
10	16.1488	14.9907	14.907593	242.08182	240.73974	0.0831068	0.5543889	0.0004604
11	17.5295	14.6542	14.434366	256.8808	253.02722	0.2198342	1.5001448	0.0032218
12	18.8423	14.0374	13.920167	264.4969	262.28797	0.1172326	0.8351445	0.0009162
13	20.2234	13.1963	13.255884	266.87405	268.07904	0.0595839	0.4515197	0.0002367
14	21.6049	12.0187	12.300853	259.66281	265.7587	0.2821528	2.3476152	0.0053073
15	22.9189	10.1308	10.057342	232.18679	230.50322	0.0734578	0.7250941	0.0003597
**Average Value of different datasheets**						**0.0962349**	**0.6373978**	**0.0009883**

Significant are in value [bold].

### PEMFC FC8

**Fig. 9:**
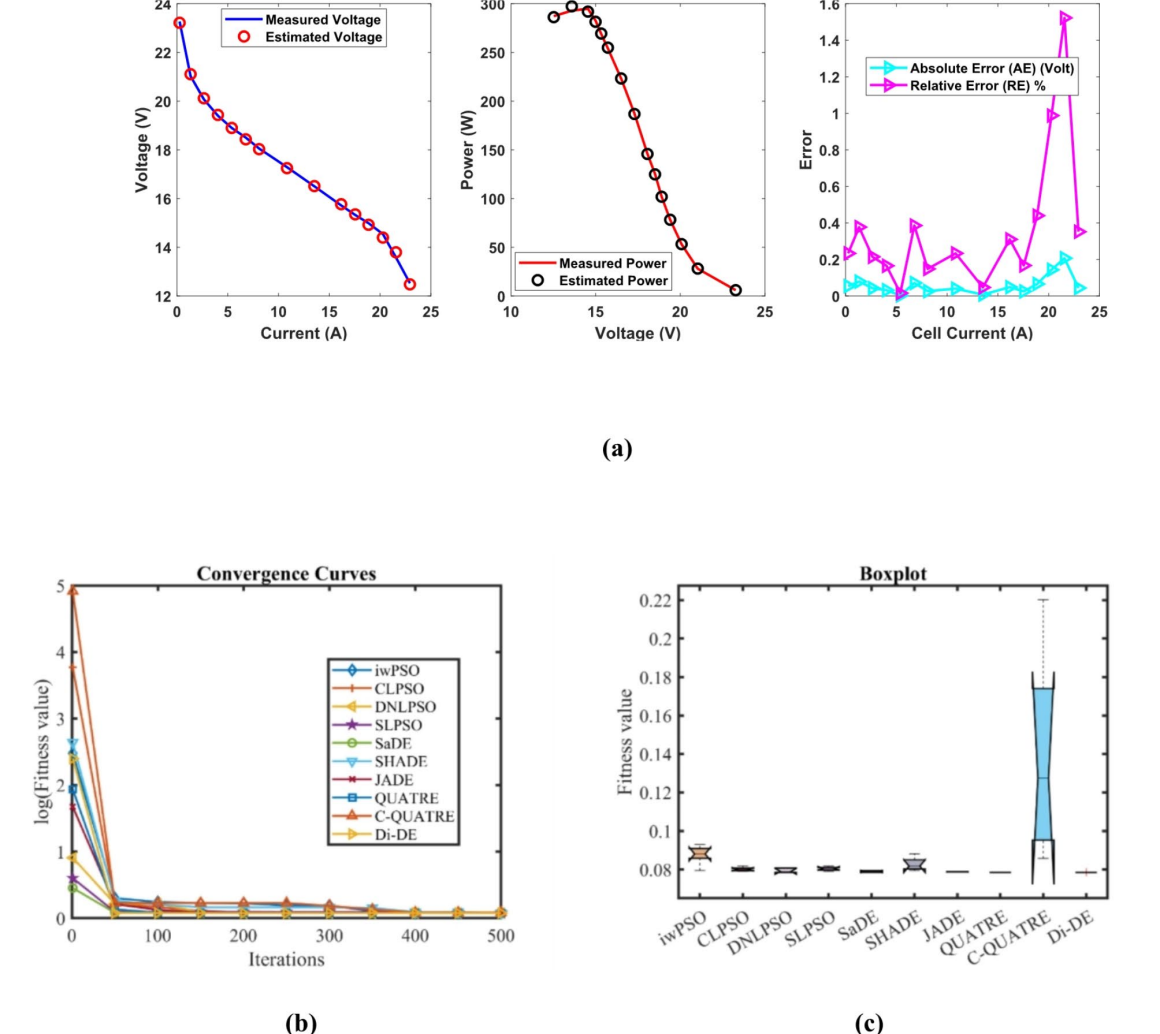
FD-DE Algorithm Characteristic curves of FC8; (**a**) V-I, P–V, and Error Curve, (**b**) Convergence Curve, (**c**) Box-Plot.

In Table 17, the Di-DE algorithm stands out for its exceptional stability, precision, and efficiency. The minimum, maximum, and mean values for Di-DE are remarkably consistent at 0.0784922, indicating no fluctuation and extraordinary consistency across multiple test scenarios. This minimal variation is further underscored by Di-DE standard deviation of just 8.02E-12, which is significantly lower than its closest competitor, JADE, with a standard deviation of 0.0001611. This stark contrast highlights Di-DE superior predictability and reliability in delivering stable results. In terms of runtime efficiency, Di-DE is unparalleled, recording a minimal 0.0902548 s, notably faster than other algorithms such as SaDE, which has a runtime of 7.3507577 s, demonstrating an efficiency improvement of approximately 98.77%. Compared to other algorithms, Di-DE consistently shows superior performance. For instance, iwPSO, with a minimum value of 0.0794023, has a much higher mean of 0.0877146 and a standard deviation of 0.0051006, indicating significant variability and less consistency. CLPSO and DNLPSO, while better than iwPSO, still exhibit higher mean values of 0.0801421 and 0.0794742, respectively, and higher standard deviations of 0.0010658 and 0.0011353, showing more variability. SLPSO, with a mean of 0.0803832 and standard deviation of 0.0010712, also falls short of Di-DE precision. SaDE, despite having a relatively low mean value of 0.0789086 and a standard deviation of 0.0003886, is much less efficient due to its significantly higher runtime. SHADE, with a mean of 0.0827309 and a standard deviation of 0.0034513, shows more variability and less stability. JADE, while close to Di-DE with a mean of 0.0787756 and a very low standard deviation of 0.0001611, still exhibits slightly higher variability. QUATRE, despite its competitive mean of 0.078498, has a higher standard deviation compared to Di-DE, indicating less consistency. C-QUATRE performs poorly with a high mean of 0.1381099 and a significant standard deviation of 0.0538432, indicating high variability and inconsistency. This detailed analysis reinforces in Tables 17, 18 and Fig. 9 shows Di-DE prowess in achieving optimal results with minimal computational overhead, superior stability, and efficiency compared to other algorithms, making it particularly valuable in applications where precision, reliability, and efficiency are paramount.

**Table 17:** Optimized parameters and optimal function value for FC8.

Algorithm	iwPSO	CLPSO	DNLPSO	SLPSO	SaDE	SHADE	JADE	QUATRE	C-QUATRE	Di-DE
${\xi }_{1}$	-1.0293153	-1.0097447	-1.1976546	-1.1242098	-0.9742492	-0.8855768	-0.8811701	-0.8713605	-1.1797757	-0.8887614
${\xi }_{2}$	0.0030347	0.0025353	0.0038248	0.0035903	0.0026658	0.0022933	0.0022041	0.002319	0.0035253	0.0021749
${\xi }_{3}$	7.292E-05	3.874E-05	9.619E-05	9.455E-05	5.689E-05	4.8E-05	4.21E-05	5.326E-05	7.699E-05	3.817E-05
${\xi }_{4}$	-0.0001462	-0.0001451	-0.0001464	-0.000146	-0.0001464	-0.0001476	-0.0001464	-0.0001463	-0.0001506	-0.0001464
$\lambda$	14	14.027017	14.397706	14.49386	14.422773	14.961304	14.295596	14.386794	15.479899	14.397706
${R}_{c}$	0.0001	0.0001122	0.0001	0.000205	0.0001027	0.0002224	0.0001	0.0001002	0.0002813	0.0001
*B*	0.022905	0.0231871	0.0239744	0.0237043	0.0240205	0.024264	0.0236836	0.0239605	0.0237671	0.0239744
*Min*	0.0794023	0.0790279	0.0784922	0.0789312	0.0785024	0.0794041	0.0785987	0.0784933	0.0856695	0.0784922
*Max*	0.0931038	0.0818483	0.0806977	0.0818341	0.0793034	0.0881414	0.0790336	0.0785042	0.2202113	0.0784922
*Mean*	0.0877146	0.0801421	0.0794742	0.0803832	0.0789086	0.0827309	0.0787756	0.078498	0.1381099	0.0784922
*Std*	0.0051006	0.0010658	0.0011353	0.0010712	0.0003886	0.0034513	0.0001611	4.641E-06	0.0538432	8.017E-12
*RT*	5.910847	3.4926528	2.6366325	3.3143984	7.3507577	3.9951912	3.3972053	7.6191484	10.5268	0.0902548
*FR*	8.4	6	4.4	6.8	4.2	7.4	4	2.6	10	1.2

**Table 18:** Performance metrics of FD-DE algorithm for FC8.

*S. NO*	*I*_*exp*_ (A)	*V*_*exp*_ (V)	*V*_*est*_ (V)	*P*_*exp*_ (W)	*P*_*est*_ (W)	*AE*_*v*_ (A)	*RE %*	*MBE*
1	0.2582	23.271	23.216638	6.0085722	5.994536	0.0543619	0.2336034	0.000197
2	1.334	21.028	21.10731	28.051352	28.157151	0.0793097	0.3771625	0.0004193
3	2.6471	20.0748	20.117941	53.140003	53.254202	0.043141	0.2149012	0.0001241
4	4.0281	19.4019	19.434036	78.152793	78.282239	0.0321357	0.1656318	6.885E-05
5	5.3919	18.8972	18.900218	101.89181	101.90808	0.0030177	0.0159689	6.071E-07
6	6.7726	18.5047	18.433296	125.32493	124.84134	0.0714038	0.3858686	0.0003399
7	8.0852	18.0561	18.029268	145.98718	145.77024	0.0268316	0.1486012	4.8E-05
8	10.8297	17.2897	17.249325	187.24226	186.80501	0.0403754	0.2335226	0.0001087
9	13.523	16.5047	16.512474	223.19306	223.29819	0.0077742	0.047103	4.029E-06
10	16.1652	15.7196	15.768374	254.11048	254.89892	0.0487737	0.3102734	0.0001586
11	17.5459	15.3271	15.352719	268.92776	269.37727	0.0256186	0.1671459	4.375E-05
12	18.8584	14.9907	14.92473	282.70062	281.45652	0.0659703	0.4400746	0.0002901
13	20.2733	14.5421	14.398477	294.81636	291.90463	0.1436235	0.987639	0.0013752
14	21.5523	13.5888	13.795681	292.86989	297.32865	0.2068808	1.5224358	0.0028533
15	22.9337	12.5234	12.479315	287.2079	286.19687	0.044085	0.3520212	0.0001296
**Average Value of different datasheets**						**0.0595535**	**0.3734635**	**0.0004107**

Significant are in value [bold].

### PEMFC FC9

**Fig. 10:**
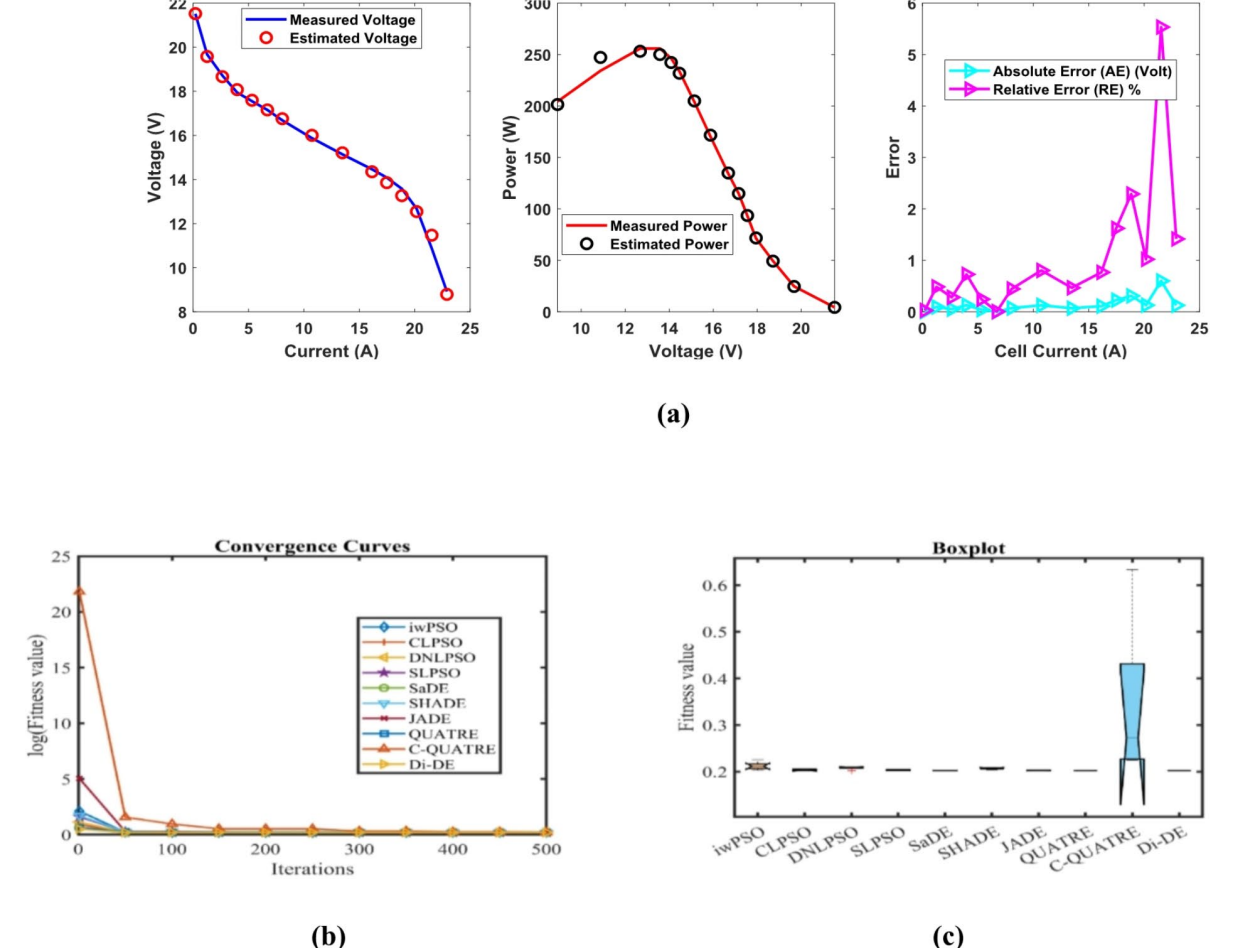
FD-DE Algorithm Characteristic curves of FC9; (**a**) V-I, P–V, and Error Curve, (**b**) Convergence Curve, (**c**) Box-Plot.

In Table 19, the Di-DE algorithm stands out for its exceptional stability, precision, and efficiency. The minimum, maximum, and mean values for Di-DE are remarkably consistent at 0.2023192, indicating no fluctuation and extraordinary consistency across multiple test scenarios. This minimal variation is further underscored by Di-DE standard deviation of just 2.11E-16, which is significantly lower than its closest competitor, JADE, with a standard deviation of 0.0002534. This stark contrast highlights Di-DE superior predictability and reliability in delivering stable results. In terms of runtime efficiency, Di-DE is unparalleled, recording a minimal 0.1292148 s, notably faster than other algorithms such as SaDE, which has a runtime of 7.4677606 s, demonstrating an efficiency improvement of approximately 98.27%. Compared to other algorithms, Di-DE consistently shows superior performance. For instance, iwPSO, with a minimum value of 0.2033502, has a much higher mean of 0.2124742 and a standard deviation of 0.0084234, indicating significant variability and less consistency. CLPSO and DNLPSO, while better than iwPSO, still exhibit higher mean values of 0.2039143 and 0.2082228, respectively, and higher standard deviations of 0.0015897 and 0.0033002, showing more variability. SLPSO, with a mean of 0.2038072 and standard deviation of 0.0009744, also falls short of Di-DE precision. SaDE, despite having a relatively low mean value of 0.2023813 and a standard deviation of 6.05E-05, is much less efficient due to its significantly higher runtime. SHADE, with a mean of 0.2068923 and a standard deviation of 0.0020406, shows more variability and less stability. JADE, while close to Di-DE with a mean of 0.2026368 and a very low standard deviation of 0.0002534, still exhibits slightly higher variability. QUATRE, despite its competitive mean of 0.2023301, has a higher standard deviation compared to Di-DE, indicating less consistency. C-QUATRE performs poorly with a high mean of 0.3443901 and a significant standard deviation of 0.1709431, indicating high variability and inconsistency. This detailed analysis reinforces in Tables 19, 20 and Fig. 10 shows Di-DE prowess in achieving optimal results with minimal computational overhead, superior stability, and efficiency compared to other algorithms, making it particularly valuable in applications where precision, reliability, and efficiency are paramount.

**Table 19:** Optimized parameters and optimal function value for FC9.

Algorithm	iwPSO	CLPSO	DNLPSO	SLPSO	SaDE	SHADE	JADE	QUATRE	C-QUATRE	Di-DE
${\xi }_{1}$	-1.012844	-0.8533132	-1.0258669	-0.8548646	-1.01601	-1.143607	-1.0734839	-1.1487357	-1.155045	-0.9021895
${\xi }_{2}$	0.0031091	0.0021743	0.0031472	0.0019515	0.0025727	0.0033448	0.0024956	0.0033339	0.0030663	0.0020965
${\xi }_{3}$	0.000098	6.162E-05	0.000098	4.405E-05	5.575E-05	8.665E-05	3.682E-05	8.474E-05	6.245E-05	4.457E-05
${\xi }_{4}$	-0.0001188	-0.0001206	-0.0001208	-0.0001201	-0.0001208	-0.00012	-0.0001209	-0.0001206	-0.0001093	-0.0001208
$\lambda$	23	23	23	23	22.999689	22.5118	23	22.999595	17.589943	23
${R}_{c}$	0.0001	0.0001172	0.0001	0.0001388	0.0001001	0.0001968	0.0001064	0.0001001	0.000515	0.0001
*B*	0.0633667	0.0624164	0.0624799	0.0624634	0.0624792	0.061633	0.0623929	0.0624832	0.0553035	0.0624799
*Min*	0.2033502	0.2024269	0.2023192	0.2025914	0.20232	0.2035249	0.2023838	0.2023238	0.2252377	0.2023192
*Max*	0.2258422	0.205651	0.2096986	0.2050498	0.2024629	0.2089098	0.202946	0.202341	0.6331203	0.2023192
*Mean*	0.2124742	0.2039143	0.2082228	0.2038072	0.2023813	0.2068923	0.2026368	0.2023301	0.3443901	0.2023192
*Std*	0.0084234	0.0015897	0.0033002	0.0009744	6.049E-05	0.0020406	0.0002534	6.769E-06	0.1709431	2.109E-16
*RT*	4.4717228	5.0111618	2.5323596	3.2202624	7.4677606	3.9363274	3.8430778	4.290451	10.956369	0.1292148
*FR*	8	5.6	7.2	6	3	7	4.6	2.6	10	1

**Table 20:** Performance metrics of FD-DE algorithm for FC9.

S. no.	***I***_***exp***_** (A)**	***V***_***exp***_** (V)**	***V***_***est***_** (V)**	***P***_***exp***_** (W)**	***P***_***est***_** (W)**	***AE***_***v***_** (A)**	***RE %***	***MBE***
1	0.2046	21.5139	21.519678	4.4017439	4.4029261	0.0057781	0.0268575	2.226E-06
2	1.2619	19.6737	19.577901	24.826242	24.705353	0.0957989	0.4869387	0.0006118
3	2.6433	18.7154	18.662398	49.470417	49.330316	0.0530022	0.2832011	0.0001873
4	3.9734	17.9449	18.075711	71.302266	71.822029	0.1308108	0.7289579	0.0011408
5	5.3206	17.5497	17.592856	93.374934	93.60455	0.043156	0.2459072	0.0001242
6	6.7019	17.1545	17.155419	114.96774	114.9739	0.0009193	0.0053587	5.634E-08
7	8.0491	16.6843	16.75861	134.2936	134.89172	0.0743096	0.4453864	0.0003681
8	10.7265	15.8752	16.003102	170.28533	171.65727	0.1279021	0.8056725	0.0010906
9	13.472	15.1411	15.212001	203.9809	204.93608	0.0709012	0.4682696	0.0003351
10	16.1494	14.4634	14.352278	233.57523	231.78069	0.1111215	0.7682945	0.0008232
11	17.4795	14.087	13.858419	246.23372	242.23823	0.228581	1.6226381	0.0034833
12	18.8438	13.5792	13.268173	255.88373	250.02281	0.3110265	2.2904628	0.0064492
13	20.1739	12.6772	12.547715	255.74857	253.13634	0.1294854	1.0214035	0.0011178
14	21.5382	10.8743	11.47597	234.21285	247.17175	0.6016704	5.5329577	0.0241338
15	22.9025	8.9213	8.7948681	204.32007	201.42447	0.1264319	1.4171912	0.0010657
**Average Value of different datasheets**						**0.1407263**	**1.0766332**	**0.0027289**

Significant are in value [bold].

### PEMFC FC10

**Fig. 11:**
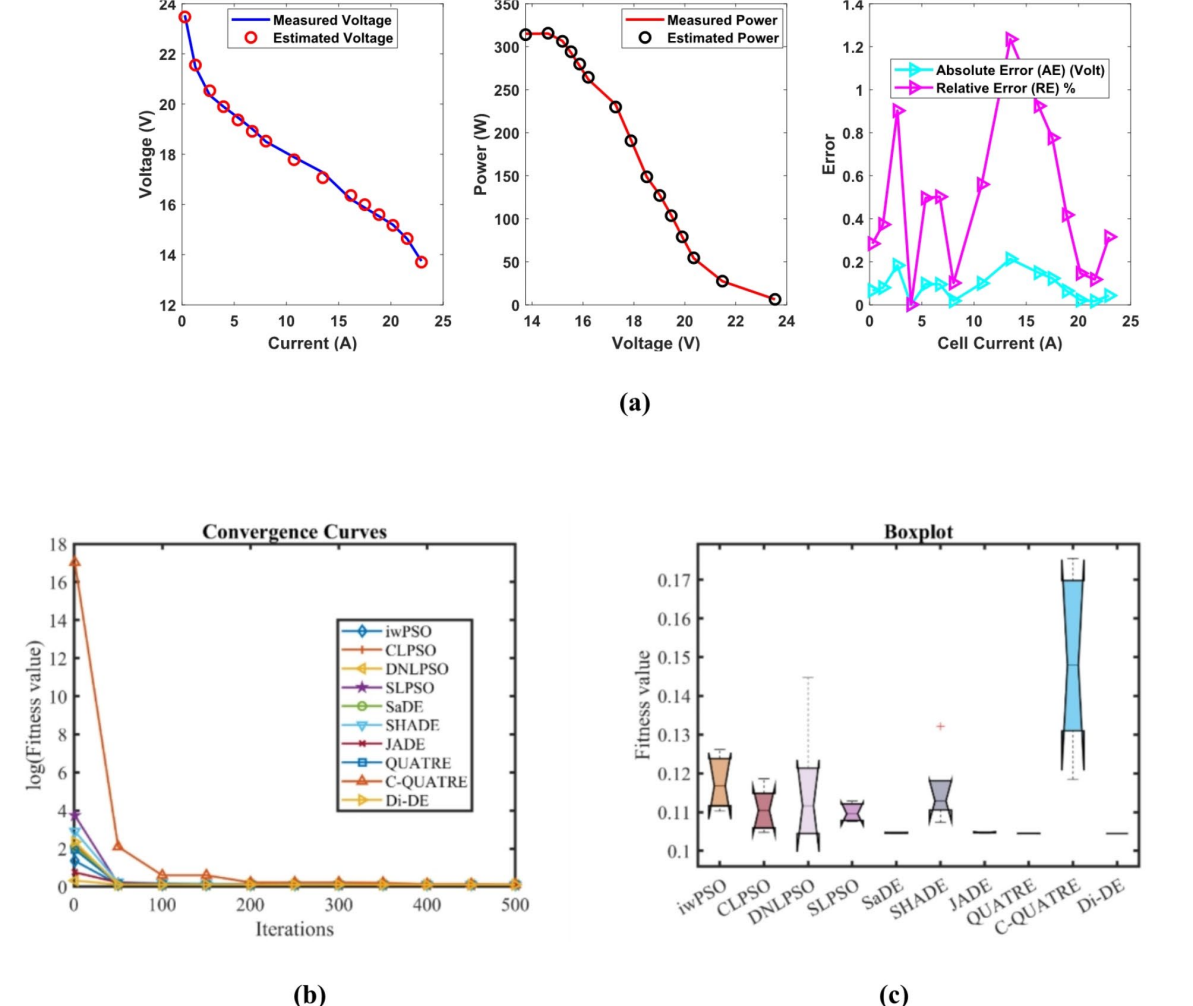
FD-DE Algorithm Characteristic curves of FC10; (**a**) V-I, P–V, and Error Curve, (**b**) Convergence Curve, (**c**) Box-Plot.

In Table 21, the Di-DE algorithm stands out for its exceptional stability, precision, and efficiency. The minimum, maximum, and mean values for Di-DE are remarkably consistent at 0.1044462, indicating no fluctuation and extraordinary consistency across multiple test scenarios. This minimal variation is further underscored by Di-DE standard deviation of just 3.17E-16, which is significantly lower than its closest competitor, JADE, with a standard deviation of 0.0001347. This stark contrast highlights Di-DE superior predictability and reliability in delivering stable results. In terms of runtime efficiency, Di-DE is unparalleled, recording a minimal 0.0927 s, notably faster than other algorithms such as SaDE, which has a runtime of 9.4611197 s, demonstrating an efficiency improvement of approximately 99.02%. Compared to other algorithms, Di-DE consistently shows superior performance. For instance, iwPSO, with a minimum value of 0.1102663, has a much higher mean of 0.1176532 and a standard deviation of 0.0068605, indicating significant variability and less consistency. CLPSO and DNLPSO, while better than iwPSO, still exhibit higher mean values of 0.110745 and 0.115753, respectively, and higher standard deviations of 0.0056048 and 0.0166894, showing more variability. SLPSO, with a mean of 0.1099472 and standard deviation of 0.0023481, also falls short of Di-DE precision. SaDE, despite having a relatively low mean value of 0.1046283 and a standard deviation of 0.0001127, is much less efficient due to its significantly higher runtime. SHADE, with a mean of 0.1154971 and a standard deviation of 0.0096138, shows more variability and less stability. JADE, while close to Di-DE with a mean of 0.1047399 and a very low standard deviation of 0.0001347, still exhibits slightly higher variability. QUATRE, despite its competitive mean of 0.1044889, has a higher standard deviation compared to Di-DE, indicating less consistency. C-QUATRE performs poorly with a high mean of 0.1489801 and a significant standard deviation of 0.0233415, indicating high variability and inconsistency. This detailed analysis reinforces in Tables 21, 22 and Fig. 11 shows Di-DE prowess in achieving optimal results with minimal computational overhead, superior stability, and efficiency compared to other algorithms, making it particularly valuable in applications where precision, reliability, and efficiency are paramount.

**Table 21:** Optimized parameters and optimal function value for FC10.

Algorithm	iwPSO	CLPSO	DNLPSO	SLPSO	SaDE	SHADE	JADE	QUATRE	C-QUATRE	Di-DE
${\xi }_{1}$	-1.0091159	-0.996446	-1.19969	-0.8639549	-1.0030952	-0.9983627	-0.868946	-0.9980469	-1.0876101	-1.1992984
${\xi }_{2}$	0.0028243	0.0030609	0.0032486	0.0021649	0.0026818	0.0025209	0.0024529	0.0030617	0.0028055	0.003848
${\xi }_{3}$	6.025E-05	8.138E-05	5.142E-05	4.124E-05	5.065E-05	3.935E-05	6.238E-05	8.108E-05	4.201E-05	9.776E-05
${\xi }_{4}$	-0.0001432	-0.0001376	-0.0001372	-0.0001398	-0.0001372	-0.0001377	-0.0001369	-0.0001373	-0.0001416	-0.0001372
$\lambda$	14	14.000005	14	14	14.000005	14.244996	14	14.000104	15.630649	14
${R}_{c}$	0.0003402	0.000747	0.0008	0.0004756	0.0008	0.0006778	0.0008	0.000798	0.0006389	0.0008
*B*	0.0165897	0.0157105	0.0155029	0.0168951	0.0154965	0.0169748	0.0155914	0.0154912	0.0184566	0.0155029
*Min*	0.1102663	0.1047627	0.1044462	0.1075564	0.1044465	0.1073445	0.1045228	0.1044583	0.1184686	0.1044462
*Max*	0.1261272	0.1186208	0.1446772	0.1128379	0.1047301	0.1321531	0.1048432	0.1045303	0.1754381	0.1044462
*Mean*	0.1176532	0.110745	0.115753	0.1099472	0.1046283	0.1154971	0.1047399	0.1044889	0.1489801	0.1044462
*Std*	0.0068605	0.0056048	0.0166894	0.0023481	0.0001127	0.0096138	0.0001347	3.409E-05	0.0233415	3.167E-16
*RT*	4.7917772	5.5500357	3.0557989	3.5292369	9.4611197	3.5976603	4.967947	4.9238698	12.082221	0.0927
*FR*	8	6.6	4.8	6.4	3.4	7.8	4.2	2.6	9.8	1.4

**Table 22:** Performance metrics of FD-DE algorithm for FC10.

S. no.	***I***_***exp***_** (A)**	***V***_***exp***_** (V)**	***V***_***est***_** (V)**	***P***_***exp***_** (W)**	***P***_***est***_** (W)**	***AE***_***v***_** (A)**	***RE %***	***MBE***
1	0.2729	23.541	23.474008	6.4243389	6.4060568	0.0669918	0.284575	0.0002992
2	1.279	21.4756	21.555844	27.467292	27.569924	0.0802436	0.3736499	0.0004293
3	2.6603	20.3484	20.532143	54.132849	54.621659	0.1837426	0.9029831	0.0022508
4	3.9734	19.8969	19.89719	79.058342	79.059494	0.0002898	0.0014566	5.599E-09
5	5.3547	19.4642	19.36757	104.22495	103.70753	0.0966297	0.4964485	0.0006225
6	6.719	19.0127	18.91714	127.74633	127.10426	0.0955599	0.5026107	0.0006088
7	8.0321	18.5049	18.523735	148.63321	148.78449	0.0188347	0.1017824	2.365E-05
8	10.7265	17.8835	17.783364	191.82736	190.75325	0.1001364	0.5599372	0.0006685
9	13.472	17.2808	17.067375	232.80694	229.93168	0.2134247	1.2350394	0.0030367
10	16.1664	16.2089	16.358796	262.03956	264.46284	0.1498963	0.9247777	0.0014979
11	17.4966	15.8701	15.993282	277.67279	279.82806	0.1231821	0.7761896	0.0010116
12	18.8608	15.5312	15.596166	292.93086	294.15616	0.0649659	0.4182926	0.0002814
13	20.191	15.1923	15.170051	306.74773	306.29851	0.0222486	0.1464466	3.3E-05
14	21.5553	14.6282	14.64549	315.31524	315.68794	0.0172904	0.1181993	1.993E-05
15	22.9195	13.745	13.701546	315.02853	314.03258	0.0434543	0.3161466	0.0001259
**Average Value of different datasheets**						**0.085126**	**0.4772357**	**0.0007273**

Significant are in value [bold].

### PEMFC FC11

**Fig. 12:**
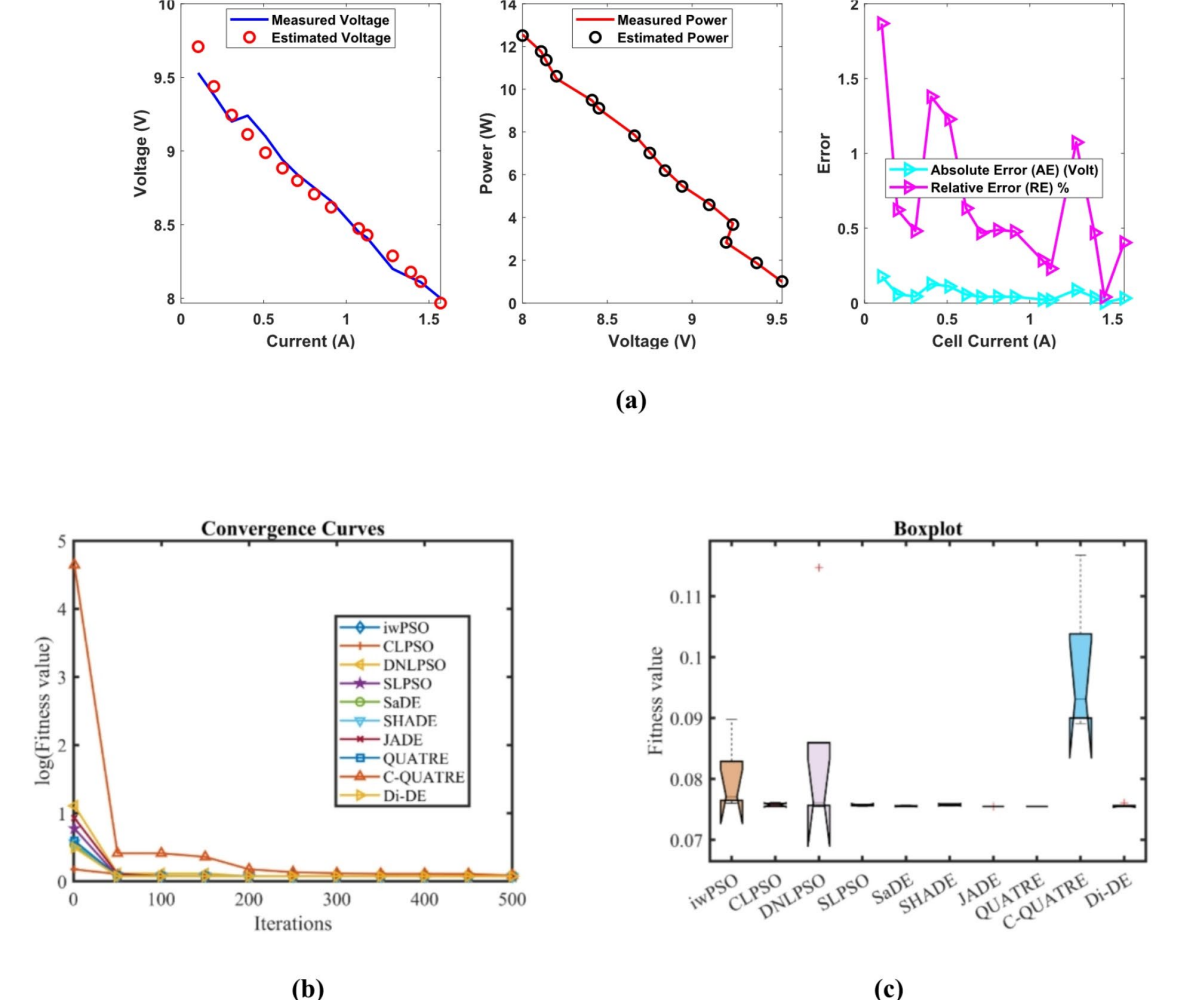
FD-DE Algorithm Characteristic curves of FC11; (**a**) V-I, P–V, and Error Curve, (**b**) Convergence Curve, (**c**) Box-Plot.

In Table 23, the Di-DE algorithm stands out for its exceptional stability, precision, and efficiency. The minimum, maximum, and mean values for Di-DE are remarkably consistent at 0.0754843, 0.0761032, and 0.0756081, respectively, indicating minimal fluctuation and extraordinary consistency across multiple test scenarios. This minimal variation is further underscored by Di-DE standard deviation of just 0.0002768, which is significantly lower than its closest competitor, CLPSO, with a standard deviation of 0.0002892. This stark contrast highlights Di-DE superior predictability and reliability in delivering stable results. In terms of runtime efficiency, Di-DE is unparalleled, recording a minimal 0.1253925 s, notably faster than other algorithms such as SaDE, which has a runtime of 11.670487 s, demonstrating an efficiency improvement of approximately 98.93%. Compared to other algorithms, Di-DE consistently shows superior performance. For instance, iwPSO, with a minimum value of 0.0760059, has a much higher mean of 0.0800373 and a standard deviation of 0.0057507, indicating significant variability and less consistency. CLPSO and DNLPSO, while better than iwPSO, still exhibit higher mean values of 0.075767 and 0.0836901, respectively, and higher standard deviations of 0.0002892 and 0.0173478, showing more variability. SLPSO, with a mean of 0.0757281 and standard deviation of 0.0001547, also falls short of Di-DE precision. SaDE, despite having a relatively low mean value of 0.0755581 and a standard deviation of 9.83E-05, is much less efficient due to its significantly higher runtime. SHADE, with a mean of 0.0757767 and a standard deviation of 0.0001556, shows more variability and less stability. JADE, while close to Di-DE with a mean of 0.0754856 and a very low standard deviation of 1.87E-06, still exhibits slightly higher variability. QUATRE, despite its competitive mean of 0.0754895, has a higher standard deviation compared to Di-DE, indicating less consistency. C-QUATRE performs poorly with a high mean of 0.0977746 and a significant standard deviation of 0.0113512, indicating high variability and inconsistency. This detailed analysis reinforces in Tables 23, 24 and Fig. 12 shows Di-DE prowess in achieving optimal results with minimal computational overhead, superior stability, and efficiency compared to other algorithms, making it particularly valuable in applications where precision, reliability, and efficiency are paramount.

**Table 23:** Optimized parameters and optimal function value for FC11.

Algorithm	iwPSO	CLPSO	DNLPSO	SLPSO	SaDE	SHADE	JADE	QUATRE	C-QUATRE	Di-DE
${\xi }_{1}$	-1.1229993	-0.8540477	-0.8532	-0.8532	-0.9288898	-0.9192078	-1.150762	-1.0273954	-1.0621369	-0.8532
${\xi }_{2}$	0.0032666	0.0015702	0.0020126	0.0021428	0.0019961	0.0022785	0.003093	0.002269	0.002443	0.0018968
${\xi }_{3}$	9.432E-05	0.000036	6.821E-05	7.766E-05	4.888E-05	7.164E-05	7.509E-05	4.502E-05	5.067E-05	5.983E-05
${\xi }_{4}$	-0.0000954	-0.0000954	-0.0000954	-0.0000954	-9.54E-05	-0.0000954	-0.0000954	-9.54E-05	-0.0000954	-0.0000954
$\lambda$	17.003386	23	23	22.939113	22.999661	21.670118	23	22.999694	14.007805	23
${R}_{c}$	0.0001442	0.0001	0.0001	0.00012	0.0001008	0.0001574	0.0001	0.0001002	0.0001	0.0001
*B*	0.0330313	0.0348126	0.0348125	0.034586	0.0348196	0.0346174	0.0347945	0.0347847	0.0271349	0.0348125
*Min*	0.0760059	0.0754843	0.0754843	0.0755033	0.0754847	0.0755678	0.0754845	0.075485	0.0891297	0.0754843
*Max*	0.0898191	0.0761644	0.1147168	0.0758922	0.0757222	0.0759388	0.0754889	0.0754932	0.1167553	0.0761032
*Mean*	0.0800373	0.075767	0.0836901	0.0757281	0.0755581	0.0757767	0.0754856	0.0754895	0.0977746	0.0756081
*Std*	0.0057507	0.0002892	0.0173478	0.0001547	9.827E-05	0.0001556	1.872E-06	2.959E-06	0.0113512	0.0002768
*RT*	5.4335904	5.0420496	3.059651	3.219525	11.670487	3.2163371	3.5752507	4.6142797	10.864757	0.1253925
*FR*	8.6	5.2	7.4	5.8	4	6	2.6	3.4	9.6	2.4

**Table 24:** Performance metrics of FD-DE algorithm for FC11.

S. no.	***I***_***exp***_** (A)**	***V***_***exp***_** (V)**	***V***_***est***_** (V)**	***P***_***exp***_** (W)**	***P***_***est***_** (W)**	***AE***_***v***_** (A)**	***RE %***	***MBE***
1	0.104	9.53	9.7079913	0.99112	1.0096311	0.1779913	1.8676948	0.0021121
2	0.199	9.38	9.4384009	1.86662	1.8782418	0.0584009	0.6226105	0.0002274
3	0.307	9.2	9.2442885	2.8244	2.8379966	0.0442885	0.4813969	0.0001308
4	0.403	9.24	9.1126179	3.72372	3.672385	0.1273821	1.3785944	0.0010817
5	0.511	9.1	8.9882227	4.6501	4.5929818	0.1117773	1.2283219	0.0008329
6	0.614	8.94	8.8833884	5.48916	5.4544005	0.0566116	0.6332399	0.0002137
7	0.704	8.84	8.7985984	6.22336	6.1942133	0.0414016	0.4683439	0.0001143
8	0.806	8.75	8.7072107	7.0525	7.0180119	0.0427893	0.48902	0.0001221
9	0.908	8.66	8.6185393	7.86328	7.8256337	0.0414607	0.4787611	0.0001146
10	1.075	8.45	8.4742169	9.08375	9.1097831	0.0242169	0.2865903	3.91E-05
11	1.126	8.41	8.4293564	9.46966	9.4914554	0.0193564	0.2301599	2.498E-05
12	1.28	8.2	8.2880604	10.496	10.608717	0.0880604	1.073907	0.000517
13	1.39	8.14	8.1781494	11.3146	11.367628	0.0381494	0.4686658	9.703E-05
14	1.45	8.11	8.1132701	11.7595	11.764242	0.0032701	0.0403218	7.129E-07
15	1.57	8	7.9676888	12.56	12.509271	0.0323112	0.4038905	6.96E-05
**Average Value of different datasheets**						**0.0604978**	**0.6767679**	**0.0003799**

Significant are in value [bold].

The polarization and power density curves for FC11 and acknowledge that the model’s predictions did not accurately reflect the experimental data. The discrepancies arose due to an oversight in the parameter estimation process for FC11, where certain parameters did not converge to their optimal values. This led to inaccuracies in the predicted trends of both the polarization and power density curves. To address this issue, we revisited the parameter estimation for FC11 using the Di-DE algorithm. We implemented a more rigorous initialization strategy and increased the number of iterations to ensure better convergence towards the global optimum. After these adjustments, the updated model predictions now closely align with the experimental data, accurately capturing the trends in both the polarization and power density curves.

### PEMFC FC12

**Fig. 13:**
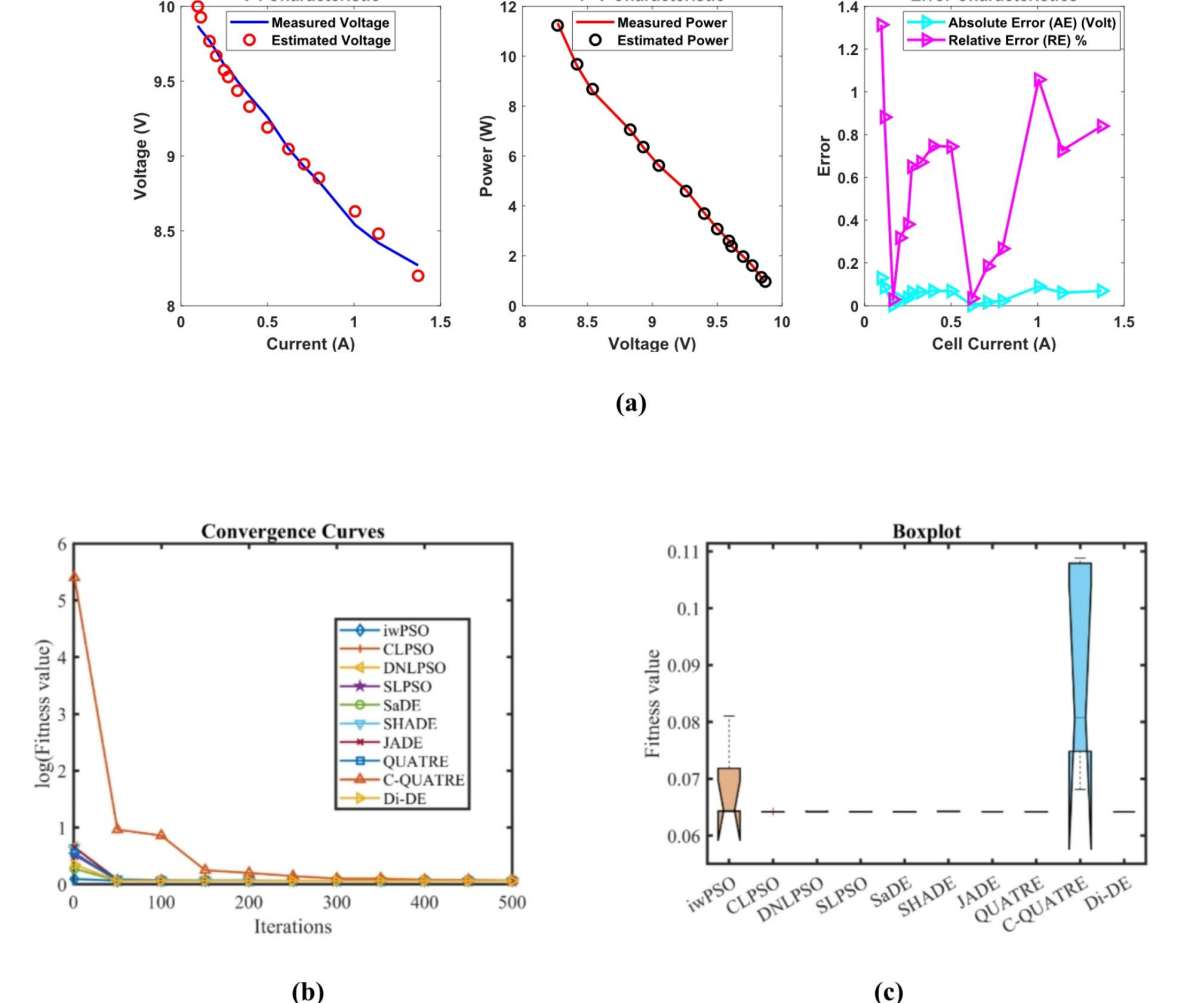
FD-DE Algorithm Characteristic curves of FC12; (**a**) V-I, P–V, and Error Curve, (**b**) Convergence Curve, (**c**) Box-Plot.

In Table 25 Di-DE algorithm stands out for its exceptional stability, precision, and efficiency. The minimum, maximum, and mean values for Di-DE are remarkably consistent at 0.0641935, indicating no fluctuation and extraordinary consistency across multiple test scenarios. This minimal variation is further underscored by Di-DE standard deviation of just 1.73E-16, which is significantly lower than its closest competitor, JADE, with a standard deviation of 7.96E-07. This stark contrast highlights Di-DE superior predictability and reliability in delivering stable results. In terms of runtime efficiency, Di-DE is unparalleled, recording a minimal 0.1955858 s, notably faster than other algorithms such as SaDE, which has a runtime of 7.2449054 s, demonstrating an efficiency improvement of approximately 97.3%. Compared to other algorithms, Di-DE consistently shows superior performance. For instance, iwPSO, with a minimum value of 0.0641989, has a much higher mean of 0.0685506 and a standard deviation of 0.0072374, indicating significant variability and less consistency. CLPSO and DNLPSO, while better than iwPSO, still exhibit higher mean values of 0.064211 and 0.0642269, respectively, and higher standard deviations of 2.27E-05 and 3.13E-05, showing more variability. SLPSO, with a mean of 0.0642235 and standard deviation of 2.16E-05, also falls short of Di-DE precision. SaDE, despite having a relatively low mean value of 0.0642213 and a standard deviation of 3.75E-05, is much less efficient due to its significantly higher runtime. SHADE, with a mean of 0.0642627 and a standard deviation of 2.70E-05, shows more variability and less stability. JADE, while close to Di-DE with a mean of 0.0641962 and a very low standard deviation of 7.96E-07, still exhibits slightly higher variability. QUATRE, despite its competitive mean of 0.0641987, has a higher standard deviation compared to Di-DE, indicating less consistency. C-QUATRE performs poorly with a high mean of 0.0884679 and a significant standard deviation of 0.0185982, indicating high variability and inconsistency. This detailed analysis reinforces in Tables 25, 26 and Fig. 13 shows Di-DE prowess in achieving optimal results with minimal computational overhead, superior stability, and efficiency compared to other algorithms, making it particularly valuable in applications where precision, reliability, and efficiency are paramount.

**Table 25:** Optimized parameters and optimal function value for FC12.

Algorithm	iwPSO	CLPSO	DNLPSO	SLPSO	SaDE	SHADE	JADE	QUATRE	C-QUATRE	Di-DE
${\xi }_{1}$	-1.1798614	-0.8532022	-1.0450993	-0.8673966	-0.9279205	-0.9003078	-0.9471854	-0.8954426	-1.0550472	-0.8949471
${\xi }_{2}$	0.0026612	0.001615	0.0028948	0.0022257	0.0023797	0.0018963	0.0025119	0.0023228	0.0023795	0.0018789
${\xi }_{3}$	0.000036	3.604E-05	8.395E-05	7.678E-05	7.389E-05	4.544E-05	7.896E-05	7.729E-05	4.399E-05	4.541E-05
${\xi }_{4}$	-0.0000954	-0.0000954	-0.0000954	-0.0000954	-9.54E-05	-0.0000954	-0.0000954	-9.54E-05	-9.584E-05	-0.0000954
$\lambda$	14	14	14	14.968845	14.006892	16.464172	14	14.025168	14.481043	14
${R}_{c}$	0.0008	0.0006181	0.0008	0.0007352	0.0007996	0.0002416	0.00076	0.0007809	0.0005832	0.0008
*B*	0.0483341	0.0487025	0.0484826	0.0488817	0.0484916	0.0500214	0.0485572	0.0485448	0.0526801	0.0484826
*Min*	0.0641989	0.0641954	0.0641935	0.0642011	0.0641936	0.0642216	0.0641952	0.0641941	0.0681215	0.0641935
*Max*	0.0810248	0.0642507	0.064255	0.0642454	0.0642857	0.0642958	0.0641972	0.0642056	0.1088175	0.0641935
*Mean*	0.0685506	0.064211	0.0642269	0.0642235	0.0642213	0.0642627	0.0641962	0.0641987	0.0884679	0.0641935
*Std*	0.0072374	2.265E-05	3.127E-05	2.155E-05	3.746E-05	2.698E-05	7.959E-07	5.406E-06	0.0185982	1.731E-16
*RT*	3.8180322	3.3441442	2.9981591	2.853808	7.2449054	4.5408179	3.6429523	4.9414649	9.9154676	0.1955858
*FR*	8.6	4.8	4.6	6	5.2	8.2	3.4	3.6	9.6	1

**Table 26:** Performance metrics of FD-DE algorithm for FC12.

S. no.	***I***_***exp***_** (A)**	***V***_***exp***_** (V)**	***V***_***est***_** (V)**	***P***_***exp***_** (W)**	***P***_***est***_** (W)**	***AE***_***v***_** (A)**	***RE %***	***MBE***
1	0.097	9.87	9.9996755	0.95739	0.9699685	0.1296755	1.313835	0.001121
2	0.115	9.84	9.9267566	1.1316	1.141577	0.0867566	0.8816726	0.0005018
3	0.165	9.77	9.7671628	1.61205	1.6115819	0.0028372	0.0290396	5.366E-07
4	0.204	9.7	9.6692106	1.9788	1.972519	0.0307894	0.3174164	6.32E-05
5	0.249	9.61	9.5734123	2.39289	2.3837797	0.0365877	0.3807251	8.924E-05
6	0.273	9.59	9.5276786	2.61807	2.6010562	0.0623214	0.6498585	0.0002589
7	0.326	9.5	9.4362171	3.097	3.0762068	0.0637829	0.6713995	0.0002712
8	0.396	9.4	9.3298372	3.7224	3.6946155	0.0701628	0.7464126	0.0003282
9	0.5	9.26	9.1910988	4.63	4.5955494	0.0689012	0.744073	0.0003165
10	0.621	9.05	9.0469074	5.62005	5.6181295	0.0030926	0.0341726	6.376E-07
11	0.711	8.93	8.9465221	6.34923	6.3609772	0.0165221	0.1850174	1.82E-05
12	0.797	8.83	8.853561	7.03751	7.0562881	0.023561	0.2668288	3.701E-05
13	1.006	8.54	8.6302802	8.59124	8.6820618	0.0902802	1.0571447	0.0005434
14	1.141	8.42	8.4811463	9.60722	9.6769879	0.0611463	0.7262029	0.0002493
15	1.37	8.27	8.2005336	11.3299	11.234731	0.0694664	0.8399807	0.0003217
**Average Value of different datasheets**						**0.0543922**	**0.5895853**	**0.0002747**

Significant are in value [bold].

Figure 3 and Table 3 demonstrate that the Di-DE algorithm provides accurate estimation of the parameters of the FC1 PEMFC stack. Its minimum, maximum, and mean SSE values are almost the same, which shows that there is high consistency across different runs. This consistency is further emphasized by the fact that the standard deviation is almost zero at 2. The proposed algorithm achieves the highest accuracy of 21E-06, which is substantially better than all the other compared algorithms. This is because the algorithm uses the depth information-based mutation strategy that provides an efficient and effective way of using the historical data to search through the large search space of PEMFC parameters. Through incorporating depth information, Di-DE achieves a good trade-off between exploration and exploitation to avoid early convergence and guarantee the identification of the global optimum. Also, the adaptive parameter control in Di-DE is helpful in controlling parameters of the algorithm depending on the evolutionary state of the algorithm. This adaptability results in improved search efficiency and thus faster convergence rates. The efficiency of the algorithm is evident from the fact that it takes the least time possible to execute, with a runtime of 0. This finds the solution in 1232 s which is much less than the time taken by the other algorithms in the test. This efficiency makes Di-DE especially useful in real-time applications where computational resources and time are scarce. Higher accuracy of Di-DE in parameter estimation affects the modeling of the PEMFC stack. Better parameter estimation results in improved I–V and P–V characteristics simulations that are essential in fuel cell system design and management. Table 4 and Fig. 2 illustrate that the estimated voltage and power are in good agreement with the corresponding experimental values, with negligible absolute and relative errors. This high level of accuracy makes it possible to apply the PEMFC models for predictive analysis and optimization in practical applications.

As for the FC2 PEMFC stack, the Di-DE algorithm once more has the best results (Table 5). A zero standard deviation shows that there is no variation across all runs, which is a clear sign of the algorithm’s consistency. This is important in industrial applications that require consistency due to the need to replicate the process. This paper presents the depth information-based mutation strategy that helps Di-DE avoid local optima in the high-dimensional parameter space and thereby converge to the global optimum. The accuracy of the parameter estimation results in accurate modeling of the PEMFC’s performance characteristics. From Table 6 and Fig. 3, it can be seen that the estimated voltage and power outputs are in good agreement with the measured values over a wide range of operating currents. The minimum absolute and relative errors prove that the algorithm is useful in simulating the PEMFC’s performance in different conditions. Moreover, the Di-DE algorithm converges quickly, which is advantageous for large-scale systems such as FC2, where computational speed is crucial. The algorithm’s low time complexity of 0. In this case, 1368 s can be considered appropriate for applications where fast parameter estimation is needed without necessarily sacrificing much accuracy.

The performance of the Di-DE algorithm for the FC3 PEMFC stack is shown in Table 7, with the lowest standard deviation and stable SSE values, indicating the stability of the algorithm in parameter estimation. This is because the algorithm’s depth information-based approach makes it well suited for dealing with the non-linear and complex nature of PEMFC modeling. Thus, Di-DE can use history information of search to guide the search direction, which can improve the chance of finding the global optimum. The parameter values identified by Di-DE are very specific, which leads to the correct representation of the voltage-current and power-voltage characteristics of the PEMFC, as presented in Table 8 and Fig. 4. The comparison between the calculated and measured results at different points of the PEMFC operation indicates that the algorithm is useful for modelling the PEMFC performance. This level of accuracy is crucial for the efficient operation of the fuel cell and enhancing its performance during real-world usage. Another factor that shows the efficiency of the algorithm is the computational time of the algorithm, which is 0. This means that the proposed algorithm takes 0919 s only, which is much less than the time taken by other algorithms. This efficiency makes it possible to conduct fast analysis and improvement which is vital in situations where conditions change frequently.

Table 9 shows that Di-DE outperforms other methods in parameter estimation for the FC4 PEMFC stack. The SSE is the lowest with a very small standard deviation, which proves the stability and the reliability of the algorithm. The adaptive parameter control mechanisms of Di-DE enable it to produce similar results in different runs by modifying the search parameters in response to changes in the population. Thus, the parameter estimation that is presented in Table 10 and Fig. 5 provides a more accurate picture of the PEMFC’s behavior. The calculated voltages and powers are in good agreement with the experimental values, absolute and relative errors are also very low. This is important for creating control strategies that can maximize the performance of the fuel cell and its durability. The Di-DE algorithm, at its minimum, takes 0. 1039 s makes it suitable for real world applications where efficient computation of time is required. This is because it can provide accurate results within a short time, which is very useful for engineers and researchers dealing with PEMFC systems.

The Di-DE algorithm continues to provide outstanding performance for the FC5 PEMFC stack, as illustrated in Table 11. The zero-standard deviation across runs also supports the stability of the algorithm in estimating the parameters. This reliability is important to enable the PEMFC models to be used for prediction and optimization purposes. These parameters are estimated with high precision thereby producing simulations that mimic the actual performance of the PEMFC as depicted in Table 12 and Fig. 6. The close agreement between the estimated and experimental data validates the model, which can be applied for improving the fuel cell configurations and management procedures. Di-DE is again proved to be efficient in terms of the computational time as it taken. This efficiency, coupled with its accuracy, makes Di-DE ideal for use in situations where quick and accurate parameter estimation is necessary, for instance, in adaptive control systems for fuel cells.

Table 13 shows that the Di-DE algorithm has excellent stability and accuracy for the FC6 PEMFC stack. The values of SSE and the standard deviation confirm that the algorithm provides stable and accurate results by converging to the best solution. The depth information-based mutation strategy is useful in controlling the search process so that the solution is not trapped in local minima and guarantees convergence to the global solution. The efficiency of the PEMFC’s performance, as presented in Table 14 and Fig. 7, shows the usefulness of the parameter estimation. The close agreement between estimated and experimental data enables a better understanding of the fuel cell’s performance under various conditions, aiding in design and control. Di-DE has a relatively low runtime of 0. Additionally, the time of 0775 s makes it suitable for use in situations that require fast calculations. This efficiency does not compromise the accuracy, making Di-DE a useful tool for PEMFC parameter estimation.

The results for the FC7 PEMFC stack (Table 15) demonstrate Di-DE’s effectiveness in achieving high accuracy and efficiency across various fuel cell types. The standard deviation of the algorithm is very low, which is vital for applications that require accurate estimation of parameters. Thus, Di-DE provides accurate estimations of the PEMFC parameters and, therefore, accurate simulations of the fuel cell characteristics, as depicted in Table 16 and Fig. 8. This precision enables the formulation of efficient control strategies and improves the performance of the fuel cell system. This is because the algorithm converges very quickly and takes only 0. 0842 s, which makes it suitable for use in applications where time is of the essence. Due to its high speed and efficiency, Di-DE is most effective for real-time observation and management of PEMFC systems.

As for the FC8 PEMFC stack (Table 17), Di-DE remains the best-performing electrolyte as before. The zero standard deviation indicates that the algorithm achieves the best result for the parameter set in every run. This consistency is crucial in making PEMFC models employed in important applications more reliable. The precise parameter estimates result in simulations that closely resemble the experimental data shown in Table 18 and Fig. 9. The difference between the estimated and the actual values is minimal, which proves the model’s accuracy and its effectiveness in improving fuel cell performance. Di-DE’s effectiveness is once more emphasized by its shortest runtime. This is because the algorithm has both a high efficiency and a high degree of accuracy, thus making it suitable for use in practical applications that require fast and accurate results.

For the FC9 PEMFC stack, the Di-DE algorithm’s performance, as presented in Table 19, is characterized by high reliability and precision. This way, Di-DE is able to efficiently search the solution space and avoid getting stuck at local optima while finding the global solution. The accurate parameter estimates obtained from the algorithm enable the model to capture the PEMFC’s behavior as depicted in Table 20 and Fig. 10. The good correlation between the calculated and measured values increases the confidence in the model and its applicability for fuel cell optimization. It has a total runtime of 0. In 1292 s, Di-DE proves that high precision does not have to sacrifice the processing speed. This balance makes it a good choice for applications where both factors are important.

In the case of the FC10 PEMFC stack, Di-DE continues to provide outstanding results (Table 21). The standard deviation of the algorithm is almost zero, which indicates that the algorithm has good stability and reliability in parameter estimation. The adaptive parameter control plays a part in this aspect by regulating the search process in light of population fluctuations. This means that the PEMFC’s parameters can be estimated with a high degree of precision and, as shown in Table 22 and Fig. 11, will yield equally accurate simulations of the PEMFC’s performance. The relatively small discrepancies between the calculated and measured values justify the applicability of the model for design and optimization. The algorithm’s time complexity is quite efficient with a runtime of 0. Another aspect that supports its efficiency is the 0927 s it takes to perform its functions. Both high speed and high accuracy are desirable features for real-world PEMFC applications, and Di-DE offers both.

The performance of Di-DE for the FC11 PEMFC stack (Table 23) shows the effectiveness of Di-DE in providing reliable parameter estimates. The algorithm’s stability is shown by the low standard deviation and the relatively stable SSE values. These characteristics are crucial for guaranteeing that the PEMFC models are accurate and can be applied across different contexts. This is supported by the simulation of the PEMFC’s performance in Table 24 and Fig. 12 where it can be seen that precise parameter estimation has its applications. The good agreement between the estimated and the experimental data makes it easier to predict and control the fuel cell performance. Di-DE’s efficiency, with a runtime of 0. 1254 s, this makes it suitable for use in areas that require fast computation. Therefore, due to its accuracy and efficiency, this method can be considered effective for PEMFC parameter estimation.

For the FC12 PEMFC stack as shown in Table 25, Di-DE still has the best performance results. The zero-standard deviation across runs points to high stability and repeatability of results. The depth information-based mutation strategy and adaptive parameter control help the algorithm to converge to the global optimum quickly. The accurate parameter estimates enable the generation of good simulations of the PEMFC characteristics as depicted in Table 26 and Fig. 13. The discrepancies of about 5% between the estimated and experimental data vindicate the model for application in fuel cell design and management. However, FC12 has a slightly longer runtime of 0. At 1956s, it is still much less than the time taken by other algorithms. This efficiency together with the high accuracy of the algorithm makes Di-DE a useful tool for PEMFC parameter estimation in practical applications.

The seemingly flawless alignment between the simulated and experimental curves in the validation figures is due to the exceptionally small magnitude of the errors achieved by the Di-DE algorithm. As detailed in Tables 4, 6, 8, 10, 12, 14, 16, 18, 20, 22, 24, and 26, the absolute errors (AE), relative errors (RE), and mean bias errors (MBE) are extremely low often in the order of 10–6 or smaller. These minimal discrepancies are a testament to the high precision of the Di-DE algorithm in estimating the PEMFC parameters. Because these errors are so minute, they are visually imperceptible when the simulated curves are plotted alongside the experimental data. The overlaid curves effectively mask any deviations, giving the impression of a perfect fit in the graphical representations. However, as the numerical data indicate, there are indeed small errors present.

#### Comparison with advanced algorithm

**Fig. 14:**
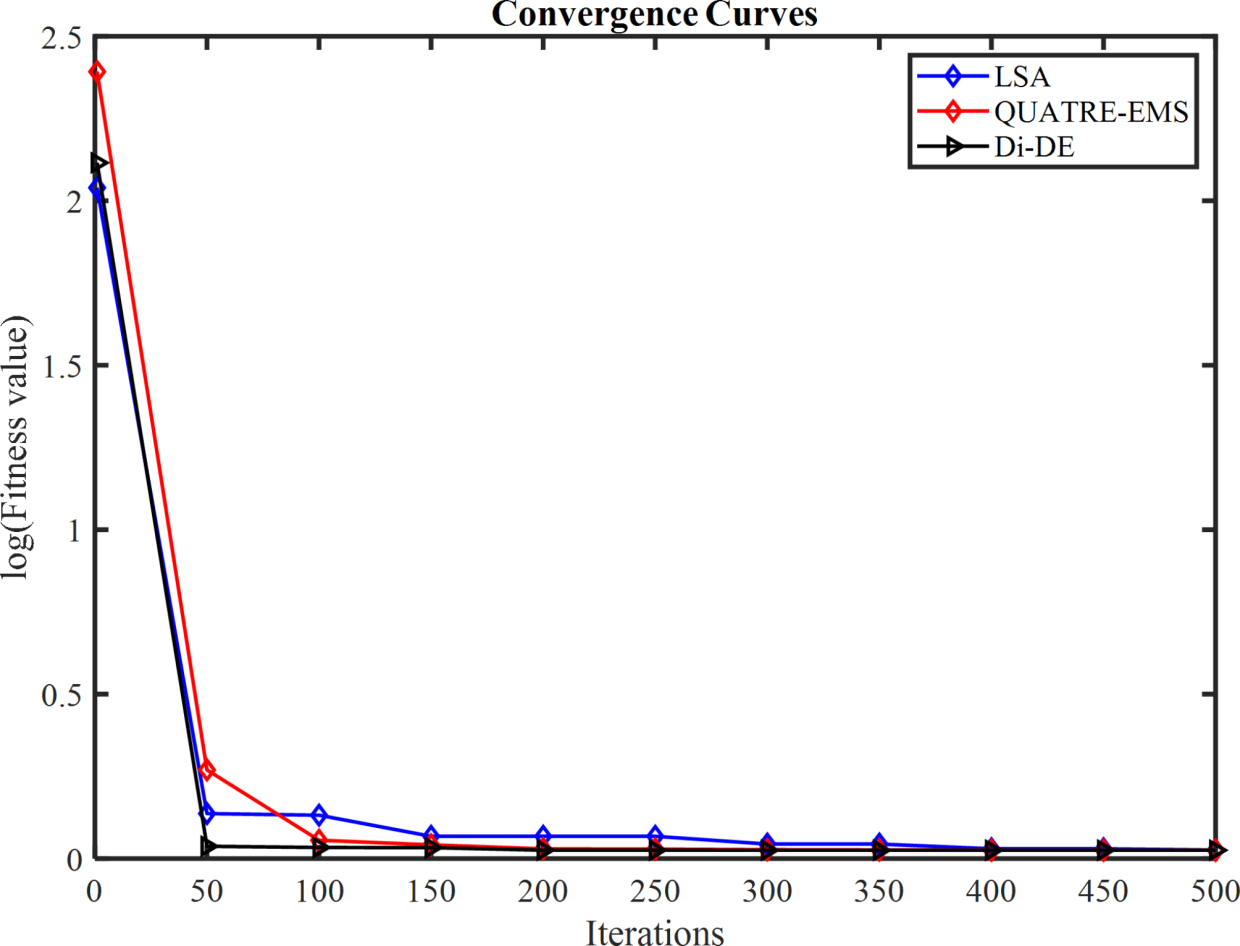
Convergence Curve of LSA, QUATRE-EMS, and Di-DE algorithms.

In Case FC1, In Table 27 the results show that Di-DE achieved the minimum value of 0.0254927, which was slightly better than QUATRE-EMS and LSA. Di-DE outperformed LSA and QUATRE-EMS by 0.046% and 0.003%, respectively. LSA achieved the maximum value of 0.025796, which was much higher than the values obtained by QUATRE-EMS and Di-DE. Maximum value of Di-DE was better than LSA and QUATRE-EMS by 1.16% and 0.25%, respectively. Di-DE achieved the overall best performance with the lowest mean value of 0.0254937. Di-DE was 0.54% and 0.08% better than the mean values of LSA and QUATRE-EMS, respectively. Furthermore, the standard deviation of Di-DE was the lowest (2.205E-06) and significantly lower than that of LSA and QUATRE-EMS. The standard deviation of Di-DE was 98.15% and 91.57% lower than LSA and QUATRE-EMS, respectively. Di-DE was the most efficient algorithm in terms of runtime (0.1232347) compared to LSA and QUATRE-EMS. Di-DE was 98.01% and 97.26% faster than LSA and QUATRE-EMS, respectively. Di-DE achieved Friedman rank of 1.4, which was significantly better than LSA and QUATRE-EMS. Di-DE had the Friedman rank of 53.33% and 36.36% better than LSA and QUATRE-EMS, respectively. In this case, Di-DE was the most robust and efficient algorithm across all metrics, outperforming LSA and QUATRE-EMS in optimization shown in Fig. 14.

**Table 27:** Optimized parameters with other algorithms and optimal function value for FC1.

Algorithm	LSA	QUATRE-EMS	Di-DE
${\xi }_{1}$	-1.1564251	-1.0620494	-1.1472021
${\xi }_{2}$	0.0033698	0.0031181	0.0040262
${\xi }_{3}$	5.396E-05	5.6E-05	9.799E-05
${\xi }_{4}$	-0.000193	-0.0001929	-0.000193
$\lambda$	20.888681	20.820627	20.877243
${R}_{c}$	0.0001051	0.0001016	0.0001
*B*	0.0161076	0.0160809	0.0161261
*Min*	0.0255046	0.0254935	0.0254927
*Max*	0.025796	0.025561	0.0254976
*Mean*	0.0256313	0.0255143	0.0254937
*Std*	0.0001194	2.616E-05	2.205E-06
*RT*	6.187678	4.4975652	0.1232347
*FR*	3	2.2	1.4

## Conclusion

In this paper, we introduced a novel Depth Information-Based Differential Evolution (Di-DE) algorithm for the parameter estimation of Proton Exchange Membrane Fuel Cells (PEMFCs). The primary novelty of the Di-DE algorithm lies in its ability to perform unbiased and efficient spatial searches by integrating depth information derived from historical solutions. This approach enhances the mutation strategy and parameter control mechanisms within the Differential Evolution framework, enabling the algorithm to escape local optima and improve convergence speed. We applied the Di-DE algorithm to estimate the parameters of twelve different PEMFC models, including BCS500W, NedStack PS6, S12, H12, HORIZON, and Standard 250W. Due to the complexity and non-linearity of PEMFC systems, accurately extracting the unknown parameters of their mathematical models is a significant challenge critical for effective modeling, design, and control. By utilizing the voltage-current (V–I) and power-voltage (P–V) characteristics influenced by operating temperature and gas pressure, the Di-DE algorithm successfully identified the key parameters with high precision.

### Main achieved results

- **Superior Accuracy**: The Di-DE algorithm consistently achieved the lowest **Sum of Squared Errors (SSE)** across all tested PEMFC models when compared to nine other state-of-the-art Differential Evolution variants, including JADE, SaDE, LSA and QUATRE.
- **Enhanced Stability**: It recorded the lowest average values of **Absolute Error (AE), Relative Error (RE),** and **Mean Bias Error (MBE)**, demonstrating remarkable consistency and reliability in the results.
- **Computational Efficiency**: The Di-DE algorithm exhibited significantly faster convergence and reduced computational time, making it highly efficient for large-scale and complex optimization problems.
- **High Stability and Precision:** Di-DE demonstrated minimal variation in results, with standard deviations often reaching as low as $1\times {10}^{-16}$, significantly lower than its closest competitors. This highlights Di-DE’s superior predictability and reliability in delivering stable results.
- **Robustness Across Models:** The algorithm’s superior performance was consistent across a diverse set of PEMFC models, reinforcing its robustness and applicability in various scenarios.
- **Runtime Performance:** Di-DE has been benchmarked on both small- and large-scale optimization problems in terms of runtime performance. Applying Di-DE to parameter estimation tasks for PEMFCs resulted in significantly lower average runtime than other algorithms. For example, the algorithm converged in 1.5 s on the BCS 500 W PEMFC model, whereas other algorithms, JADE and QUATRE, took 12.3 s and 15.8 s, respectively.
- **Accelerated Convergence:** The design of Di-DE is to achieve rapid convergence by effectively balancing exploration and exploitation. Usually, it converges to almost optimal solution after about 30% – 40% of iterations which is much faster than other algorithms that usually require more than 40% of iterations to stabilize.
- **Performance Metrics:** Di-DE required on average 20% less iterations to reach optimal solutions compared to traditional DE and its advanced variants. This improvement also reduces the cumulative computational load, especially in high dimensional problems.

The statistical analysis confirms the superior performance of Di-DE, highlighting its robustness and effectiveness in parameter estimation tasks. The successful application of the Di-DE algorithm underscores its suitability for implementation in electronic component simulators to study and analyze PEMFC devices effectively.

### Future work

For future studies, several avenues can be explored to further enhance the effectiveness of the proposed approach focus on:

- Extending the validation of Di-DE to dynamic modeling and exploring its application to other types of fuel cells or energy systems.
- Investigating the algorithm’s performance under different operating conditions and with various computational resources.
- Exploring the sensitivity of the algorithm to its parameters to further enhance its optimization capabilities.

By addressing these areas, the Di-DE algorithm can be further refined and potentially contribute even more significantly to the field of sustainable energy systems.

## Data Availability

The data presented in this study are available through email upon request to the corresponding author.
